# Magnesium Ions as Modulators of Voltage-Gated and Ligand-Gated Ion Channels in Central Neurons

**DOI:** 10.3390/ijms262412152

**Published:** 2025-12-17

**Authors:** Svetolik Spasic, Marko Biorac, Nikola Jovanovic, Srdjan Lopicic, Sanjin Kovacevic, Jelena Nesovic Ostojic, Marija Stanojević

**Affiliations:** 1 Institute for Pathological Physiology “Ljubodrag Buba Mihailović”, Faculty of Medicine, University of Belgrade, 11000 Belgrade, Serbia; svetolik.spasic@med.bg.ac.rs (S.S.);; 2 Faculty of Medicine, University of Belgrade, 11000 Belgrade, Serbia

**Keywords:** magnesium, Kir channel, Ca_V_ channel, Na_V_ channel, BK channel, SK channel, NMDAR, P2XR, GABA_A_R, nAChR

## Abstract

Magnesium ions regulate synaptic and nonsynaptic neuronal excitability from intracellular (Mg^2+^*_i_*) and extracellular (Mg^2+^*_o_*) domains, modulating voltage- and ligand-gated ion channels. K+ inward rectifier (Kir) channel inward rectification arises from Mg^2+^*_i_* blocking the pore and outward K^+^ current, while Mg^2+^*_o_* targets external sites. Mg^2+^*_i_* causes voltage-dependent Ca^2+^ voltage-gated (Ca_V_) and Na^+^ voltage-gated (Na_V_) channel block while phosphorylation modulates channel activity. Mg^2+^*_o_* elicits direct voltage-dependent Ca_V_ channel block, and screens surface charge, and in Na_V_ channels reduces conduction and may cause depolarization by quantum tunneling across closed channels. Mg^2+^*_i_* is an allosteric large conductance Ca^2+^-activated K^+^ (BK) channel activator, binding to low-affinity sites to alter Ca^2+^ and voltage sensitivity but reduces small conductance Ca^2+^-activated K^+^ (SK) channels’ outward K^+^ current and induces inward rectification. N-Methyl-D-aspartate receptor (NMDAR) channels are inhibited by Mg^2+^*_i_* binding within the pore, while Mg^2+^*_o_* stabilizes excitability through voltage-dependent block, Mg^2+^*_o_* forms Mg-ATP complex modifying purinergic P2X receptor (P2XR) channel affinity and gating and directly blocks the pore. Mg^2+^*_o_* reduces gamma-aminobutyric acid type A receptor (GABA_A_R) channel Cl^−^ current amplitude and augments susceptibility to blockers. Mg^2+^*_o_* and Mg^2+^*_i_* block nicotinic acetylcholine receptor (nAChR) channels through voltage-dependent pore binding and surface charge screening, impeding current flow and altering gating.

## 1. Introduction

Magnesium (Mg) is a mineral micronutrient bioessential for human health and wellbeing. In our cells and tissues, it serves many structural and metabolic functions, as well as important electrophysiological roles. Magnesium ion (Mg^2+^) is an electrolyte present in all our body fluids with a dominant intracellular distribution. In the brain, Mg^2+^ is ubiquitous, but more abundant in the cerebrospinal and interstitial fluid than in the blood, reflecting high neuronal demand for Mg^2+^. Within the nerve cells, Mg^2+^ distribution is compartmentalized, with specific localization in different cellular structures: the cytoplasm, cell nucleus, mitochondria, and endoplasmic reticulum (ER). Since mitochondria serve as the site of cellular respiration and major adenosine triphosphate (ATP) synthesis, they also store the highest concentration of Mg^2+^ in the cell. They have the capacity to actively accumulate Mg^2+^ and release it in response to various biological stimuli. In eukaryotic cells, mitochondrial Mg^2+^ dynamics and Mg^2+^ permeation across the inner mitochondrial membrane are facilitated by specific Mg^2+^-sensitive channels [1]. Several other transporters and exchangers have been identified on the cell membrane and organelles’ membranes responsible for maintaining cellular Mg^2+^ homeostasis [2]. Studies focusing on the effects of intracellular Mg^2+^ (Mg^2+^*_i_*) on cell energy metabolism report several mechanisms of modulation of oxidative phosphorylation by Mg^2+^ in the mitochondrial matrix. The impact of Mg^2+^*_i_* on cell respiration is described to be manifold, with a resulting enhancement of mitochondrial ATP synthesis [3].

Mg^2+^ ions distributed throughout the cell and across various compartments serve many distinct roles, critical for neuronal health and activity. Magnesium is essential for various nerve cell functions, including enzyme activation, protein synthesis, genome stabilization, cell cycle regulation, energy metabolism, regulation of biochemical pathways, signaling pathways, and modulation of membrane ion transport mechanisms. Little is known that other than intracellular Ca^2+^ ion (Ca^2+^*_i_*), Mg^2+^*_i_* can also be considered an intracellular messenger, as Mg^2+^*_i_* relays signals within the cell between different compartments. This, in fact, refers to the free, unbound form of Mg^2+^*_i_*, also known as the ionized Mg^2+^ fraction, physiologically present in low concentrations in the cytosol of the resting cell (0.5–1.2 mmol/L) [4]. Cytosolic Mg^2+^ fluctuations occur upon stimulation by various extra- or intracellular stimuli, whereby Mg^2+^*_i_* concentrations ([Mg^2+^]*_i_*) can change dynamically. Mg^2+^_i_ can rapidly be mobilized from the cellular stores or enter from the extracellular fluid, and act as a signal transducer and cellular regulator to perform many roles. It can directly control the activity of various target cell molecules: proteins, enzymes, nucleotides, ATP, etc., usually by binding to their specific motifs [5].

The concentration of free, ionized Mg^2+^ in human brain tissue fluid measures approximately 0.5 mmol/L (as determined by ^31^P phosphorus magnetic resonance spectroscopy), which is similar to that in nerve cell cytosol, adequate to ATP levels and energy demands of central neurons. In the blood and all our body fluids, Mg^2+^ exists in two main forms: the bound form (comprising the protein-bound Mg^2+^ and Mg^2+^ complexed with different ligands, including ATP), and the free form (ionized Mg^2+^ or unbound Mg^2+^). These three Mg^2+^ fractions (three states in which Mg^2+^ can be found) exist in equilibrium, but with constant dynamic Mg^2+^ shifts between them. It is the ionized Mg^2+^ fraction (iMg^2+^) that is free to exert regulatory Mg^2+^ effects (metabolic, immune, and electrophysiological). Only the iMg^2+^ is available for biological actions, being unbound and exchangeable between the compartments of body fluids. Although frequent Mg^2+^ shifts occur, equilibrium is maintained in a manner that both intra- and extracellular concentrations of iMg^2+^ are physiologically stable and approximately equal. Assessing the Mg^2+^*_i_* and extracellular (outer) Mg^2+^ (Mg^2+^*_o_*) levels in the brain provides better insights into neuronal Mg^2+^ homeostasis and its involvement in cell signaling, electrophysiology, and bioenergetics [6]. From both the intra- and the extracellular side of the cell membrane, Mg^2+^ serves the role of a regulatory cation, known for its overall stabilizing effect on electrically excitable membranes. Its homeostasis helps maintain the physiological functions of central neurons in a complex manner and even more tightly than in other types of excitable cells. Many of its actions couple cell metabolism to its electrical activity [4].

Regular neural and muscular electrical excitability and conductivity are essential to enable proper vital body functions such as sensorineural alertness, consciousness, and other nervous system functions, heart action, blood circulation, and respiration. Other than the traditionally considered electrolytes (Na^+^, K^+^, Ca^2+^, and Cl^−^), Mg^2+^ is also required for their maintenance, although it is frequently neglected when testing for the routine blood serum ionogram. Clinical implications of imbalances of Mg^2+^ metabolism are numerous. Systemic Mg^2+^ homeostasis with stable concentrations of Mg^2+^ ([Mg^2+^]) in the whole body is required for human health. On the other hand, chronic Mg^2+^ deficiency is associated with chronic low-grade hyperexcitability [4]. Because of its many pathophysiological effects, such as Mg^2+^ may be a biomarker of poor outcomes and unwanted cardiovascular events, including death, angina, myocardial infarction, heart failure, arrhythmias, and stroke [7]. In the fields of neurology, neuropsychiatry, neurosurgery, and neurorehabilitation, it is implicated in a number of conditions, like epilepsy, anxiety, depression, migraine and tension headache, Alzheimer’s disease, Parkinson’s disease, stroke recovery, cerebral vasospasm, insomnia, restless leg syndrome, etc. [8,9]. In order to better understand the potency of Mg^2+^ deficiency to mediate neuropathophysiological mechanisms of these diseases, one must comprehend well the basic electrophysiological effects of Mg^2+^ on a molecular level. This review aims to present some of them, affecting major voltage-gated and ligand-gated ion channels of central neurons.

## 2. Intracellular and Extracellular Magnesium as Modulators of Ion Channel Function

Ion channels are integral membrane proteins that allow the selective passage of ions across cellular membranes. Their activity is tightly regulated by voltage changes, ligand binding, and intracellular signaling molecules. Magnesium, the most abundant divalent intracellular cation, plays a dual role in modulating ion channels depending on its localization. Magnesium ions can affect both voltage-gated and ligand-gated ion channels, from both the extracellular and intracellular sides of the membrane. Due to their double positive charge, Mg^2+^ ions in aqueous solutions and all body fluids carry a large surface charge that strongly attracts dipoles of water molecules. In this hydrated state, as Mg^2+^(aq), they have a large ionic radius, which does not prevent them from being attracted to charged binding sites in the pores of ion channels more specific for some other ions (such as Ca^2+^, K+, or Na^+^). However, their double hydration shell is not easy to shed, making it very difficult for Mg^2+^(aq) ions to pass through the narrow pores of ion channels in biological membranes. This makes Mg^2+^ a suitable modulator of activity in a number of different ion channels. Once Mg^2+^ ions bind within the channel pore, it becomes challenging for the competing cations to displace it, and channel permeation becomes blocked [10]. Over time, it has been shown that Mg^2+^ influences ion channel function through several mechanisms, such as electrostatic interactions with channel pores or gating domains, allosteric modulation of channel conformation, surface charge shift that alters membrane potential sensing, or competition with other cations (Ca^2+^, Na^+^) for binding or permeating the channel pore.

**Figure 1:**
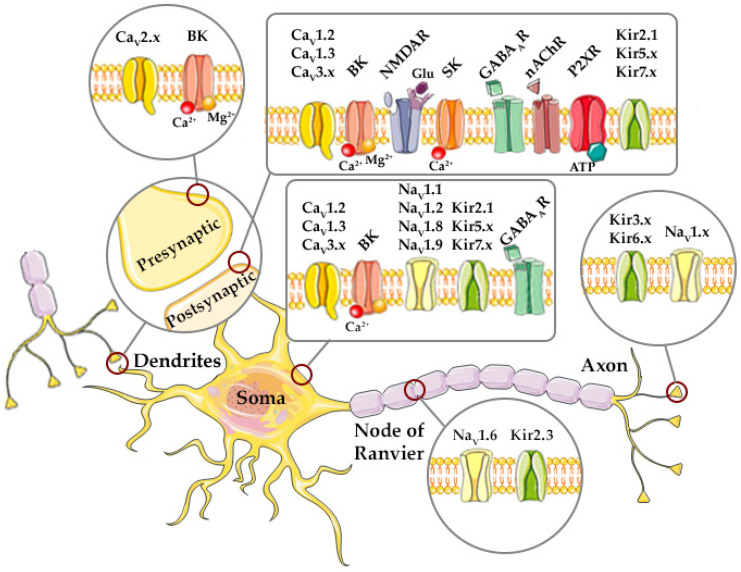
Localization of major voltage-gated and ligand-gated ion channels in neurons shown to be regulated by Mg^2+^. Kir—inward rectifier K^+^ channel, Ca_V_—voltage-gated Ca^2+^ channel, Na_V_—voltage-gated Na^+^ channel, BK—large conductance Ca^2+^-activated K^+^ channel, SK—small conductance Ca^2+^-activated K^+^ channel, P2XRs—purinergic P2X receptors, NMDAR—N-methyl-D-aspartate Receptor, Glu—glutamate, GABA_A_R—type A Gamma-Amino Butyric Acid Receptor, nAChR—nicotinic ACh receptor. Adapted from Servier Medical Art (https://smart.servier.com).

Magnesium acts as an important determinant in physiological and pathophysiological conditions, and the mechanisms of its action that underlie therapeutic potential are of great interest to basic scientists and practicing clinicians. However, we are still lacking complete knowledge regarding the precise mechanisms of Mg^2+^ effects on excitable membranes. Understanding structural interactions of Mg^2+^ with different ion channels, ramifications of these interactions, and their effects on channel functionality, is thereafter crucial for our comprehension of the mechanisms underlying alterations of functions of excitable membranes associated with magnesium imbalances, as well as the effects of potential therapeutic applications of magnesium in the conditions of magnesium deficiency. Therefore, we aim here to present an overview of research data on the interactions between Mg^2+^*_i_* and Mg^2+^*_o_* with some of the most important voltage-gated and ligand-gated ion channels shown to be affected by Mg^2+^ (Figure 1).

### 2.1. Voltage-Gated Ion Channels Regulated by Mg^2+^

Voltage-gated channels (VGCs) are transmembrane proteins that mediate ion flux across the cell membrane in response to changes in membrane potential. They play a pivotal role in electrical signaling processes in the body–neurotransmission, muscle contraction, and hormone secretion. The canonical action of these channels arises from an interplay of their components and functions, (a) channel voltage sensing is mediated by special voltage-sensing domains (VSD), (b) conformational changes of channel pores shift between pore opening and pore closing, (c) channels actively transition from open state to closed state, while channel inactivation results from a state of a channel already closed but which cannot be reopened temporarily, and finally (d) ion selectivity of some VGCs comes from specific ion-selectivity filter regions within the channel pore. Magnesium ions, normally present in the extracellular and intracellular fluids (both cytosolic and subcellular compartments), can affect the function of VGCs. Perturbations in the [Mg^2+^], even within their physiological range, can augment changes in channel activity. These effects are more prominently seen with [Mg^2+^]*_i_* changes and with larger variations in Mg^2+^*_o_* concentration ([Mg^2+^]*_o_*) changes. Here we present the findings concerning Mg^2+^ effects on several voltage-gated ion channels, inward rectifier K^+^ channels, voltage-gated Ca^2+^ channels, voltage-gated Na^+^ channels, and large conductance Ca^2+^-activated K^+^ channels, primarily in nerve cells, but also in some other cell types (microglia, astrocytes, cardiomyocytes, vascular smooth muscle cells, and endothelial cells).

#### 2.1.1. Mg^2+^ Effects on Inward Rectifier Potassium (Kir) Channels

Kir channels allow influx of K^+^ ions into the cells and conduct a more inward K^+^ current during hyperpolarizing membrane potentials (negative to the K^+^ equilibrium potential), while restricting outward K^+^ current amid membrane depolarization [11,12,13]. These channels are tetramers, and each subunit contains two transmembrane (TM) helices (the outer, TM1 helix and the inner, TM2 helix), linked by the pore-forming region (H5) that carries the ion-selectivity filter and large N-terminal (amino, NH_2_^−^) and C-terminal (carboxy, COOH-) regions that constitute the cytoplasmic domain (CTD). The Kir channel structure is missing a VSD [14].

Functional studies suggest that K^+^ ions interact with certain charged residues in the Kir channel CTD, while traversing the extended channel pore composed of the CTD pore and TM pore [15]. The selectivity filter at the extracellular region of the channel pore can also serve as a gating element [16]. Though not being voltage-gated, Kir channels are of interest due to a highly voltage-dependent block of outward K^+^ currents, elicited by Mg^2+^*_i_* [11,12,17,18,19,20]. This group of K^+^-selective ion channels is critical for maintaining the resting membrane potential, regulating neuronal and myocyte excitability, and facilitating K^+^ homeostasis in the nervous system. For example, Kir1.1 channels act as K^+^ transporters, regulating their excretion and electrolyte balance as they are expressed in renal tubules [14], but their function in the hippocampus and cortex still remains unknown [21]. Kir2.1 channels are expressed diffusely in the whole brain, on neuron somata and dendrites, endothelium, heart muscle, vascular smooth muscles and skeletal muscles, Kir2.2 channels throughout the forebrain and strongly in the cerebellum, but also in the myocardium and skeletal muscles, Kir2.3 channels mainly in the forebrain, olfactory bulb, cardiomyocytes and skeletal myocytes and in the microvilli of Schwann cells at nodes on Ranvier, while Kir2.4 are found in the cranial nerve motor nuclei in the midbrain, pons, and medulla. Since being expressed in excitable tissues, the Kir2.x family helps maintain resting membrane potential and contributes to cardiac excitability. Kir3.1, Kir3.2, and Kir3.3 channel types are expressed throughout the brain, some such as Kir3.2 in homomeric form or as Kir3.1/3.2, Kir 3.2/3.3 heteromers, and mediate inhibitory neurotransmission. Kir4.1 and Kir5.1 channels are expressed on astrocytes in the brain either in Kir4.1 homomeric or Kir4.1/5.1 heteromeric form, respectively, and they mediate K^+^ buffering, which is important for the control of neuronal function in the central nervous system (CNS), but also in the spinal cord, retina, and *stria vascularis* in the inner ear. Kir6.x channels are expressed in all muscle cell types, the brain, and the pancreas, and act as metabolic sensors, while Kir7.1, found in the retina, contributes to ion transport and visual function [14,21].

##### Effects of Mg^2+^*_i_* on Kir Channels

Inward rectification of Kir channels is considered to result from an endogenous effect of Mg^2+^ binding to the channel from the cytoplasmatic, intracellular side, as registered in isolated murine cardiomyocytes [11,14,22], murine erythroleukaemia (MEL) cells expressing Kir1 channels [23], or modeled *in silico* [15]. At resting membrane potential level, electrostatic forces repel Mg^2+^ from the channel pore, allowing for inward K^+^ conduction, while membrane depolarization allows Mg^2+^*_i_* to flow into the Kir channel pore, physically blocking it and inhibiting the efflux of K^+^ ions, thus stabilizing membrane potential in neurons and glial cells [15,21]. This competitive blockage of Kir channels by Mg^2+^*_i_* at depolarizing membrane potentials is crucial for the magnitude of the outward K^+^ current and K^+^ balance within the cell. Additionally, this block allows astrocytes to uptake an excess of extracellular K^+^ ions to prevent hyperexcitability during high neuronal activity [14]. Mg^2+^*_i_* produces a rapid block of outward K^+^ current in bovine vascular endothelial cells, but there seems to be an internal additional voltage-dependent gating mechanism free of Mg^2+^ influence, leading to closure of Kir channels [24]. Mg^2+^ (0.1 mmol/L) on the cytoplasmatic side of the membrane diminishes the outward K^+^ currents through open Kir channels, which become flickery, while there is no effect of Mg^2+^*_i_* on the inward currents [12,18,19]. Electrophysiological experiments on murine ventricular cardiomyocytes show that progressive increase of [Mg^2+^]*_i_* modifies the outward K^+^ current, which evolves from an open channel current to zero-current level, as [Mg^2+^]*_i_* reaches 1 mmol/L [12,18]. Mg^2+^ ions have a diameter close to that of K^+^ ions, making it able to block the channels that permeate K^+^ and possess K^+^-binding sites [21].

Over time, it was established that Kir channel subunits possess more than one binding site for Mg^2+^. Mg^2+^*_i_* can interact with negatively charged residues of the Kir channel pore lining in the cytoplasmatic and transmembrane portion of the channel in the process of rectification [15]. Based on the sensitivity to Mg^2+^*_i_* block, Kir channels are recognized as either strong or weak inward rectifiers [17]. Electrophysiological experiments on *Xenopus* oocytes expressing various Kir channel mutants identified one of the crucial sites within the channel pore for Mg^2+^ to bind. This site is in the vicinity of a negatively charged residue, aspartate (Asp) side chain in position 171, as it is shown that substitution of Kir1.1 channel’s polar uncharged asparagine (Asn) with negatively charged Asp in this position (N171D) elicits a 20-fold stronger affinity of the channel to Mg^2+^ and blocks outward K^+^ currents [17]. Additionally, findings from the same model, expressing mutant Kir channels, reveal that a single substitution of Asp within the channel’s transmembrane M2 domain at position 172 with Asn (D172N) transforms a strong inward rectifier Kir2.1 channel into a channel with weak rectifier-like properties. Reversed substitution of Asn in position 171 with Asp (N172D) converts a weak rectifier Kir into a channel with a strong rectifier nature [25]. These findings are corroborated on MEL cells by showing that a mutation induced to a Kir2.1 channel, altering negatively charged Asp in position 172 to uncharged glutamine (Gln) (D172Q), reduces the channel’s affinity to Mg^2+^*_i_* more than five-fold [26]. Electrophysiological experiments on oocytes expressing mutant Kir channels suggest another site that is involved in channel rectification. Changing the negatively charged glutamate (Glu) in position 224 of the CTD C-terminus with non-polar glycine (Gly) or polar uncharged Gln or serine (Ser) (E224G/Q/S, respectively) drastically reduces rectification of the mutant channel. Furthermore, it is possible that the Asp residue at position 172 interacts with the Glu residue at position 224 to form a binding pocket for Mg^2+^ [27]. Multiscale modeling was utilized to investigate the path that Mg^2+^*_i_* crosses from the cytoplasm through the Kir2.1 channel pore, reaching the conclusion that these ions extensively interact with a negatively charged Glu residue in position 299 at the center of the intracellular domain, in order to block K^+^ outward current [28].

Membrane patches excised from murine cardiomyocytes [11], oocytes expressing Kir channels [29] or vascular endothelial cells [24] used to record single Kir channel currents exhibit rapid rundown of Kir activity in an internal solution containing physiological [Mg^2+^]*_i_* (~1 mmol/L), but restore channel rectification capability, while channel activity in Mg^2+^-free solution, although preserved, leads to a loss of inward rectification. Although it was initially thought that changes in intracellular K^+^ concentration ([K^+^]*_i_*) have little to no effect on the blocking action of Mg^2+^*_i_* on Kir outward currents [19], it is now known that a decrease in [K^+^]*_i_* augments the blocking affinity of Mg^2+^*_i_* on Kir1.1 expressed on *Xenopus* oocytes [30]. Additionally, as extracellular K^+^ concentration ([K^+^]*_o_*) increases, the Mg^2+^*_i_* blocking affinity for different families of Kir channels decreases, and a stronger depolarization is required to achieve the same amount of Kir blockage by Mg^2+^*_i_* [17,30].

##### Effects of Mg^2+^*_o_* on Kir Channels

Mg^2+^*_o_* can also modulate the activity of Kir channels. Electrophysiological recordings of membrane patches of vascular endothelial cells [24], oocytes expressing Kir2.1 [31], Kir2.2 [16] or Kir1.1 [20] channels exposed to increasing concentrations of Mg^2+^*_o_* demonstrate a decay of Kir channel activity, a reduction in the outward K^+^ current amplitude, and a rise in the extent of Kir channel inactivation. Removal of extracellular bivalent cations, such as Mg^2+^, reduces the extent of Kir channel voltage-dependent inhibition [16,20,24]. Presence of Mg^2+^*_o_* elicits longer periods of Kir channel closure intermittently separated by bursts of channel activity reflecting Mg^2+^ entry, association with the channel pore binding site, followed by dissociation from the site and exit from the channel [24]. Inward rectifier K^+^ channels (Kir2.1) expressed on *Xenopus* oocytes exhibit greater sensitivity to Mg^2+^*_o_*-induced voltage-dependent block than Kir2.2 and Kir2.3 channels. One of the mechanisms underlying this effect might be the interaction of Mg^2+^*_o_* with a negatively charged Glu residue at position 125 located in the extracellular loop between TM1 and pore domain (PD) of Kir2.1 channel. Substitution of Glu with Asp (E125D) preserves the sensitivity to Mg^2+^*_o_*-induced block of the mutant channel, proving that the negatively charged amino acid residue at position 125 is potentially a binding site for Mg^2+^*_o_*. Moreover, mutant Kir2.1 channel with polar uncharged Gln instead of Glu (E125Q) reduces channel sensitivity to Mg^2+^*_o_*, whereas substitution of Gln (Q126E) in mutant Kir2.2 and positively charged histidine (His) (H116E) in mutant Kir2.3 channels with Glu increases their sensitivity to Mg^2+^*_o_* block [32]. However, an increase in [K^+^]*_o_* reduces the Mg^2+^*_o_*-induced blocking effect on Kir channel activity, possibly by competing for the same binding site in the channel pore in murine cardiomyocytes [11,19], endothelial cells [24] and oocytes expressing Kir2.1 [31,32], Kir2.2 [16] or Kir1.1 [20] channels. Specific residues in the outer region of the channel could constitute a functional K^+^ sensor that alters its activity to changes in [K^+^]*_o_*. Negatively charged amino acid residues of the outer mouth of the Kir channel and the pore’s selectivity filter attract K^+^ ions, increasing their concentration in this region. Electrostatic repulsion between cations potentially repels Mg^2+^ from binding to the channel, thus reducing Mg^2+^*_o_*-induced voltage-dependent block of inward K^+^ current [16].

Finally, concerning other types of voltage-gated K^+^ (K_V_) channels, there are findings that K_V_1-K_V_3 are susceptible to increasing [Mg^2+^]*_i_* as it blocks outward K^+^ currents in a voltage-dependent manner [33,34]. Membrane depolarization can enhance Mg^2+^*_i_* blocking action of K_V_1.5 and K_V_2.1 channels to the degree that channels exhibit inward rectification in the presence of Mg^2+^*_i_* at positive membrane voltages, reminiscent of Kir channels [33]. In addition to the direct channel pore block, Mg^2+^*_i_* modulates the channel’s voltage sensor activity by screening negative cytosolic surface charges and shifts activation and inactivation to more negative membrane potentials [33,35]. Contrary to its direct channel blocking action, Mg^2+^*_i_* decreases KCNQ (K^+^ voltage-gated channel subfamily Q, K_V_7) channel currents by binding phosphatidylinositol 4,5-bisphosphate (PIP2), a membrane lipid instrumental for KCNQ channel activation, thereby leaving only a limited fraction of free PIP2 available to interact with the channel [36]. Conversely, voltage-dependent activation of EAG (ether-à-go-go, K_V_10) and hERG (human ether-à-go-go related gene, K_V_11) channels is modulated by Mg^2+^*_o_*, slowing the channel’s gating kinetics and transition to an open state as it screens negatively charged amino acid residues in the channel’s voltage-sensing region [37,38,39,40].

#### 2.1.2. Mg^2+^ Effects on Voltage-Gated Calcium (Ca_V_) Channels

In response to membrane depolarization, Ca_V_ channels mediate Ca^2+^ influx that regulates neurotransmitter release, excitability, and intracellular signaling in neurons; cardiac action potential (AP); muscle contractions; hormone secretion; and gene transcription. These channels consist of a pore-forming Ca_V_α1 subunit and auxiliary subunits that attune gating activity–an intracellular Ca_V_β-subunit (Ca_V_β1–Ca_V_β4) and a complex extracellular Ca_V_α2δ-subunit (Ca_V_α2δ1–Ca_V_α2δ4) [41,42]. The Ca_V_α1 subunit is composed of four homologous repeats (I–IV), each containing six TM helices (S1-S6), where S5 and S6 form channel PD, a loop linking S5 and S6 (p-loop) carries the Ca^2+^ selectivity filter region, while S1–S4 helices constitute channel VSD [43]. Ca_V_ channels are classified into three subfamilies. Firstly, Ca_V_1.1–Ca_V_1.4 channels are defined as L-type for their large, long-lasting currents or as dihydropyridine receptors (DHPRs) due to their sensitivity to dihydropyridine (DHP). Ca_V_1.1 channels are expressed in skeletal muscles and Ca_V_1.4 channels in the retina, while Ca_V_1.2 and Ca_V_1.3 channels are present in cardiac and vascular smooth muscle cells, sinoatrial node (SAN), neuron soma and dendrites in the brain, intestinal/bladder smooth muscle, presynaptic side in cochlear ribbon synapses, and adrenal chromaffin cells [41,42,44]. Ca_V_2.1 channels are called P-type, as they were first described and are abundant in cerebellar Purkinje neurons, and Q-type, which were initially identified in cerebellar granule cells, and help regulate cerebellar signaling [45]. Ca_V_2.2 channels or N-type (neural type) are involved in neurotransmission in autonomic synapses and sensory synapses conveying nociceptive signals. Ca_V_2.3 channels, known as R-type as they are not only resistant to DHP but mediate Ca^2+^ currents upon P/Q- and N-type channels block, are found in hippocampal and cortical neurons. The Ca_V_2 channel subfamily is dominantly expressed in presynaptic nerve terminals and mediates coupling of presynaptic membrane depolarization and neurotransmitter release. Furthermore, some Ca_V_2 channels (Ca_V_2.1 and Ca_V_2.2) in neuron somata and dendritic spines [46,47] can associate with Ca^2+^-activated K^+^ channels (presented in detail later) to shape AP repolarization and control of neuron firing frequency, by altering K^+^ conductance, while cooperation of these channels in presynaptic membrane microdomains regulates neurotransmission [48,49]. Lastly, Ca_V_3.1–Ca_V_3.3 channels, known as T-type for their tiny and transient currents, mediate SAN pace-making activity [43,50], but also neurotransmitter release in the retina, olfactory bulbs, hippocampal, and DRG neurons [42]. Orchestrating rebound excitation, Ca_V_3 channels shape oscillatory neuron activity in the cerebellum and thalamocortical circuits as well as in sensory processing [51]. High-voltage-activated (HVA) Ca^2+^ channels (Ca_V_1 and Ca_V_2) contain Ca_V_α1-, Ca_V_β-, and Ca_V_α2δ-subunits, and their activation is achieved at more depolarized membrane potentials, while low-voltage-activated (LVA) Ca^2+^ channels (Ca_V_3) are built solely of Ca_V_α1-subunits, and they can be activated by depolarizations slightly above the resting membrane potential [41,43]. Channels interact directly or indirectly with various proteins that regulate their function, modulation, and localization within the presynaptic terminal [41]. Long-lasting Ca^2+^ currents critical for excitation and neuroendocrine regulation are mediated by Ca_V_1.2 channels, while Ca_V_2.1 channels play an essential role in neurotransmitter release. Ca_V_ channels possess self-regulatory mechanisms, allowing them to change ion permeation in response to prolonged depolarization and activity or high Ca^2+^*_i_* concentration ([Ca^2+^]*_i_*), namely through voltage-dependent inactivation (VDI) and Ca^2+^-dependent inactivation (CDI). These actions are a result of the coordinated binding of Ca_V_α1 C-terminal cytoplasmatic isoleucine-glutamine (IQ) domains with a soluble Ca^2+^ sensor, calmodulin (CaM) already complexed with Ca^2+^ to induce CDI, or an interaction between the S1–S2 interdomain loop (α1-interacting domain, AID) region with auxiliary Ca_V_β-subunits to elicit VDI [43,50,52,53]. Another pivotal region for CDI is the Ca^2+^*_i_*-binding Ca_V_α1-subunit’s C-terminus EF-hand motif, a site that is in the vicinity of the IQ domain and is proposed to also mediate in the Ca_V_ channel inactivation upon Ca^2+^-CaM binding [53].

##### Effects of Mg^2+^*_i_* on Ca_V_ Channel

The concentration of Mg^2+^ can vary in pathophysiological conditions in the brain or skeletal muscle, increase in transient cardiac ischemia, or decrease in heart failure. Increase of [Mg^2+^]*_i_*, in some cases even within physiological concentration range, alters the function of Ca_V_ channels, leading to their inhibition. The underlying mechanism of inhibition is multifaceted and can be interpreted as a concomitant effect of Mg^2+^*_i_* on the direct influence on the channel’s ion permeation and Mg^2+^*_i_*-induced modulation of the channel’s gating kinetics.

Extensive electrophysiological studies on isolated murine ventricular cardiomyocytes report on a progressive reduction in peak currents through Ca_V_1.2 channels during application of [Mg^2+^]*_i_* that reaches the higher end of the physiological range and/or supraphysiological values [54,55,56]. Additionally, cardiomyocytes exhibit progressive shortening of AP duration with increasing [Mg^2+^]*_i_* used. These effects could not be abrogated by increasing levels of Ca^2+^*_o_* ([Ca^2+^]*_o_*) [54]. Variations of [Mg^2+^]*_i_* in isolated murine artery myocytes show a dichotomous response, an initial increase in Ca^2+^-and Ba^2+^-carrying inward currents through Ca_V_1.2 channels in extremely low [Mg^2+^]*_i_* conditions, presumably due to instant reduction in Mg^2+^, followed by a progressive attenuation in current amplitude as [Mg^2+^]*_i_* increases [57]. Concentration-dependent inhibitory action of Mg^2+^*_i_* is also shown in human embryonic kidney (HEK) TsA-201 cells expressing rabbit wild-type Ca_V_1.2 channels at fixed membrane potentials. Namely, increasing [Mg^2+^]*_i_* reduces Ca_V_ channel-mediated inward currents, an effect reversed by Mg^2+^*_i_* depletion [55,58]. Moreover, the inhibitory action of increasing [Mg^2+^]*_i_* on Ca_V_ channel permeation in isolated amphibian cardiac myocytes is also present, albeit more modest than in mammalian myocytes [59,60]. Concentration surpassing the physiological range; however, they elicit a pronounced voltage-dependent blocking effect on Ca_V_ channels, augmenting the rate and extent of channel inactivation. Membrane hyperpolarization undermines the Mg^2+^*_i_*-induced Ca_V_ block, promoting influx of Ca^2+^ during the channel activation phase, while depolarizing potentials allow Mg^2+^*_i_* to enter the cytoplasmatic side of the channel pore, hindering Ca^2+^ currents [61]. Voltage dependence of Ca_V_ channel activation and inactivation can be altered at extremely high and low [Mg^2+^]*_i_*, as shown in rabbit Ca_V_1.2-expressing HEK-TsA-201 cells. High [Mg^2+^]*_i_* leads to a negative shift in the voltage dependence of both channel activation and inactivation, while very low [Mg^2+^]*_i_* causes a positive shift in the voltage–conductance relationship of Ca_V_1.2 channels [58]. It is also proposed that the reduction in inward currents mediated by Ca_V_ channels can also partially be due to higher [Mg^2+^]*_i_* screening the negative charges on the intracellular membrane surface, thus modulating channel activity [58,61].

Mg^2+^*_i_* can modulate Ca_V_1.2 channel activity by interacting with the putative Ca^2+^-binding motif of 12 amino acid residues (EF-hand region) on the C-terminal domain in Ca_V_α1-subunit. Multiple studies on Mg^2+^ binding affinity to Ca_V_ channel pore demonstrate that negatively charged amino acid residues of the proximal C-terminal EF-hand motif are the possible binding sites in the Mg^2+^*_i_*-induced blocking cascade, probably competing with Ca^2+^-CaM complex binding and leading to a partial CaM displacement. Mutant rabbit Ca_V_ channels expressed on HEK-TsA-201 cells, where negatively charged Asp is substituted with either a nonpolar alanine (Ala), polar uncharged Asn or Ser, or positively charged lysine (Lys) (D1546A/N/S/K) in position 1546 of the EF-hand motif in Ca_V_α1-subunit, show decreased affinity for Mg^2+^*_i_*. Conversely, exchanging positively charged Lys for negatively charged Asp in position 1453 or 1539 (K1453D, or K1539D) of the same region, significantly increases channel pore affinity to Mg^2+^*_i_*. In comparison to cells expressing wild-type rabbit Ca_V_1.2, channel conductance decline is seen in lower [Mg^2+^]*_i_* in cells carrying mutant K1453D or K1539D Ca_V_ EF-hand motifs, while D1546A/N/S/K Ca_V_ mutants require a several-fold higher level of cytosolic Mg^2+^ to elicit the inhibitory action [58].

The interaction between the Ca_V_α1-subunit’s distal C-terminal domain (DCT) and proximal C-terminal domain (PCT) can be autoinhibitory, as it reduces coupling of charge movement to channel opening, thus leading to prominent augmentation of voltage-dependent inactivation of Ca_V_1.2 channels. Mg^2+^*_i_* can also modulate the noncovalent bond between DCT and PCT and the voltage-dependent inactivation of Ca_V_1.2-mediated currents. The binding of Mg^2+^*_i_* to the PCT EF-hand motif is necessary for the DCT to exert the autoinhibitory effect [62]. Electrophysiological examination of isolated murine ventricular cardiomyocytes and HEK-TsA-201 cells expressing mutant variants of the channel’s Ca_V_α1-subunits shows that an increase in [Mg^2+^]*_i_* not only reduces peak amplitudes of inward Ca_V_1.2-mediated currents but also increases the rate and the duration of voltage-dependent inactivation of inward currents. Presence of mutant D1546A/N/S/K EF-hand motifs in Ca_V_α1-subunits reduces VDI and Mg^2+^*_i_*-dependent inhibitory effects on Ca_V_1.2 conductance, since only higher [Mg^2+^]*_i_* can elicit the steady state inactivation of the mutant channel compared to the wild-type one. The effect that the EF-hand motif mutation exerts on the rate and extent of VDI arises from the lack of Mg^2+^*_i_* binding affinity for the mutated region of the channel, thus failing to enhance VDI frequency and degree [62]. Elevated [Mg^2+^]*_i_* in amphibian cardiac myocytes also increases the rate and the extent of Ca_V_ channel VDI [61].

Earlier experiments on amphibian cardiomyocytes shed light on another mechanism through which Mg^2+^*_i_* can modulate Ca_V_ channels’ gating kinetics. Increased Mg^2+^*_i_* levels markedly reduce inward Ca^2+^ currents through Ca_V_ channels when they are phosphorylated [59,60]. On the other hand, some postulate that inhibitory effects on Ca_V_ conductance result from modulation of channel gating properties through phosphorylation of channel residues elicited by Mg^2+^*_i_* [63]. Having in mind that Mg^2+^*_i_* can interact with protein kinases and phosphatases to modify their activity [58], the effect that Mg^2+^*_i_* exerts on Ca_V_ channels seems dual, as Mg^2+^*_i_* possibly interacts directly with Ca_V_ channels, which, in a more phosphorylated state, are more susceptible to Mg^2+^*_i_* inhibitory effect. As with amphibian myocytes, electrophysiological recordings from murine ventricular cardiomyocytes show that rising [Mg^2+^]*_i_* causes a prominent reduction in Ca^2+^ currents through Ca_V_1.2 channels conditioned to maximal phosphorylation. Changes from the Ca_V_1.2 channel’s maximal phosphorylation state attenuate the inhibitory Mg^2+^*_i_* effect on inward Ca^2+^ currents, thus giving rise to the possibility that [Mg^2+^]*_i_* can antagonize the effects of phosphorylation on channel gating kinetics [64]. Dialysis of murine ventricular cardiomyocytes with K252a, a protein-kinase inhibitor, diminishes inward Ca^2+^ current density and any inhibitory effect of Mg^2+^*_i_* on Ca_V_1.2 channel permeation, while phosphorylation of channels mediated by dialyzed cyclic adenosine monophosphate (cAMP) requires higher [Mg^2+^]*_i_* to reduce Ca_V_ channel conductance [63]. Inhibition of Ca_V_1.2 channel conduction by high [Mg^2+^]*_i_* in isolated murine cardiomyocytes is potentiated by phosphorylation of the channel by protein kinase A (PKA), but application of phosphatase 2A (PP2A) dampens Mg^2+^*_i_*-induced inhibition as Ca_V_ channels become dephosphorylated [55]. Many potential phosphorylation sites are found in Ca_V_α1-and Ca_V_β-subunits of Ca_V_1.2 channels, and the most important ones for PKA-mediated phosphorylation are Ser residues at position 1928 (S1928) in α1_C_-subunit and positions 478 and 479 (S478 and S479, respectively), of β2_A_-subunits [65]. Recordings from HEK-TsA-201 cells expressing mutated rabbit Ca_V_1.2 channels corroborate the significance of the channel’s phosphorylation state and sensitivity to Mg^2+^*_i_*-induced decrease in channel conduction. Namely, inward Ca^2+^ currents through mutant Ca_V_1.2 channels lacking PKA phosphorylation sites are minutely affected by increasing [Mg^2+^]*_i_*, which is comparable to that of PP2A application in cells carrying wild-type channels. Conversely, truncation of the channel’s α1-subunit to increase channel pore open probability leads to a strong inhibition of inward currents through mutated channels when [Mg^2+^]*_i_* is high, comparable to blocking effects seen in dephosphorylated channels under the influence of dihydropyridine agonist BayK8644, which promotes channel pore opening [55,56]. Interestingly, increasing [Ca^2+^]*_i_* also has the capacity to dampen Mg^2+^*_i_*-induced inhibitory effects regardless of the channel phosphorylation status, indicating that, other than counteracting the Ca_V_ channel phosphorylation effects and gating kinetics, Mg^2+^*_i_* probably has a direct blocking effect on channel ion permeation [64].

While existing studies clarify the intricate mechanisms behind Mg^2+^*_i_* regulation of Ca_V_1 channels, comparable direct channel-specific processes for other Ca_V_ channel subtypes, to the best of our knowledge, have not been reported.

##### Effects of Mg^2+^*_o_* on Ca_V_ Channels

While Mg^2+^*_i_* is considered to have an intricate effect on Ca_V_ channel conductance, aimed towards complex channel blocking and modulating channel activity, Mg^2+^*_o_* is considered to have the ability to directly block Ca_V_ channels. Early electrophysiological works on murine ventricular cardiomyocytes revealed that increasing [Mg^2+^]*_o_* transformed long-lasting single channel currents into bursts of brief openings, due to Ca_V_ channels’ discrete and rapid blocking and unblocking transitions. Channel blocking rate accelerates with increasing [Mg^2+^]*_o_*, and hyperpolarizing membrane potentials expedite channel opening and closing transitions as well. This gave rise to the idea that Mg^2+^*_o_* can lodge within the Ca_V_ channel pore and block the channel [66].

Mg^2+^*_o_*-induced blocking action is voltage-dependent. Hyperpolarization of membrane potential increases the degree of Mg^2+^*_o_*-induced steady state block of Ca_V_ channels [66]. Mg^2+^*_o_* can block Ca_V_ channels quite rapidly in isolated amphibian ventricular myocytes, but has little effect on channel inactivation kinetics [61]. Voltage-clamp experiments on isolated snail neurons and Na^+^ currents through Ca_V_ channels led to a suggestion that these channels have two functional regions–an external region where divalent cations bind with high-affinity to determine channel permeability to currents carried by monovalent cations, and the ion-selective filter that determines channel selectivity for different divalent cations [67].

Experiments on chick dorsal root ganglion (DRG) neurons exhibit a voltage-dependent block of Ca_V_ channels (L-and N-type) elicited by Mg^2+^*_o_* as inward Ca^2+^ and more prominently Na^+^ currents are reduced, respectively, to increasing [Mg^2+^]*_o_*. Inhibitory effect on inward currents is dominant at hyperpolarizing membrane potentials, and as the membrane becomes more positive, the blocking action declines. Upon cessation of depolarization and returning to negative membrane potentials, Ca_V_ channels are readily blocked again [68]. Given the potential clinical and experimental importance of Mg^2+^*_o_* in the hippocampus, the cellular and molecular bases for non-physiological excitability of hippocampal neurons induced by Mg^2+^ deficiency are of great practical interest. Electrophysiological findings of Mg^2+^*_o_*-induced Ca_V_ channel block are confirmed by visual observations of fluorescent probes in isolated murine hippocampal CA1 pyramidal neurons. Increase in Ca^2+^*_i_*-induced fluorescence in response to low [Mg^2+^]*_o_*, corresponds to increased inward Ca^2+^ currents through Ca_V_ channels upon removal of the Mg^2+^ block. Findings that intracellular fluorescence dissipates when [Mg^2+^]*_o_* increases or when Ca_V_1.2 channel blockers are used further prove the blocking action of Mg^2+^*_o_* surrounding the neurons. Interestingly, Mg^2+^*_i_* levels do not significantly change in response to varying [Mg^2+^]*_o_*. It is proposed that Mg^2+^*_o_* modulates Ca_V_ channels and inward currents traversing the channel pore [69].

Equivalent concentration-dependent effects of Mg^2+^*_o_* are observed in recordings of rat pheochromocytoma (PC12) cells, possessing characteristics of peripheral sympathetic nerves. Increasing [Mg^2+^]*_o_* elicits progressive attenuation of inward currents through N-and L-type Ca_V_ channels [70]. Patch clamp recordings of isolated murine mesenteric artery myocytes show that increasing concentrations of Mg^2+^*_o_* elicit progressive reduction in Ca^2+^-and Ba^2+^-carried inward currents through Ca_V_1.2 channels, with Ba^2+^ currents being more affected [57].

Apart from the blocking action on Ca_V_ channel conductance, high [Mg^2+^*_o_*] elicits surface charge screening near the mouth of the channel pore and a voltage shift in its gating kinetics [61,68]. As Mg^2+^*_o_* binds to the negatively charged Glu or Asp residues in the extracellular loop of the pore-forming α1-subunit, this reduces the effective voltage sensed by the VSD and shifts the voltage dependence of activation toward more positive potentials. This effect can decrease the amplitude of the Ca^2+^ current through Ca_V_ channels, in addition to elevating the voltage for channel activation [44,57]. Positive divalent cations can accumulate and “screen” or neutralize the negative charge of the cell surface.

#### 2.1.3. Mg^2+^ Effects on Voltage-Gated Sodium (Na_V_) Channels

The Na_V_ channels are critical for initiating and propagating APs in neurons, playing a pivotal role in neuronal excitability and signal transmission. These channels are composed of an Na_V_α-subunit with four homologous domains (I–IV), each with six TM segments (S1–S6), where S4 segment, being rich in positively charged amino acids, acts as a VSD, while S5–S6 segments with their connecting pore loop (p-loop) build the ion-conducting pore with an inactivation gate made out of regions that link III and IV domain. Auxiliary Na_V_β-subunits (Na_V_β1–Na_V_β4) compete with each other (Na_V_β1 v. Na_V_β3 or Na_V_β2 v. Na_V_β4) for association with the Na_V_α-subunit. Although not obligatory for Na_V_ channel function, they attune the activity of the Na_V_α-subunit to modulate channel kinetics, voltage sensitivity, and sensitivity to other ligands [50,71]. Na_V_1.1, 1.2, 1.3, and 1.6 channels are primarily expressed in the central nervous system (CNS), Na_V_1.7, 1.8, and 1.9 channels in the peripheral nervous system (PNS) at nociceptors, while Na_V_1.4 and Na_V_1.5 channels are the primary Na_V_ channels in the skeletal muscles and the heart, respectively [50,72]. The channels open in response to membrane depolarization, allowing the influx of Na^+^ ions.

##### Effects of Mg^2+^*_i_* on Na_V_ Channels

Electrophysiological experiments performed on murine cerebellar granule cells show that Mg^2+^*_i_* can elicit a voltage-dependent block of Na_V_ channels by binding to the channel pore selectivity filter. During membrane depolarization, Mg^2+^*_i_* ions enter the Na_V_ channel pore several times, reducing inward Na^+^ currents by half at depolarizing potential. Increase in [Mg^2+^]*_i_* augments the blocking effect of Mg^2+^*_i_* on Na_V_ channels, while Mg^2+^*_i_*-induced inhibition of Na^+^ currents is reversed by an increase in Na^+^*_i_* concentration ([Na^+^]*_i_*) or Na^+^*_o_* concentration ([Na^+^]*_o_*), unveiling a competitive nature of this blocking effect. It is estimated that the presence of Mg^2+^ in the channel pore interferes with Na^+^ flux in both directions. Increase in [Mg^2+^]*_i_* from 0 to 30 mmol/L elicits negative V_1/2_ shifts in the range of −25 mV to −29 mV, although the voltage dependence of channel activation and inactivation does not change with [Mg^2+^]*_i_* increase [73]. Experimental recordings from *Xenopus* oocytes expressing rat brain type II Na^+^ channels [74,75], mutant K226Q Na_V_ channels (Lys 226 substituted with Gln), type III Na^+^ channels, and neuron-like Na^+^ channels in chromaffin cells [75] demonstrate that Mg^2+^*_i_* markedly reduces Na^+^ currents through tested channels in a voltage- and concentration-dependent manner. Blocking effects become more prominent at more positive membrane potentials and with increasing [Mg^2+^]*_i_* in the patching pipette [75]. Examining the effects of Mg^2+^*_i_* on mutant cZ-2 Na^+^ channels known to exhibit slow and incomplete inactivation after opening demonstrates that increasing [Mg^2+^]*_i_* elicits a reduction in Na^+^ single-channel current amplitude. Since Mg^2+^*_i_* ions act as fast blockers, closing the Na^+^ channel pore completely for short time periods, they generate a flickery blocking action. Conversely, the blocking effect of Mg^2+^*_i_* on rat brain type II Na^+^ channels is augmented with an increase in [Na^+^]*_i_*. As Mg^2+^*_i_* is coupled to ATP–ADP hydrolysis, this balances the energy in the cell, so that a higher energy consumption leads to an increase in [Mg^2+^]*_i_* that modifies Na_V_ channels to decrease in Na^+^ conductance and attenuate AP firing [75].

Mg^2+^*_i_* can modulate Na_V_ channel activity through channel phosphorylation. Mg^2+^ is crucial for the function of PKA, an enzyme responsible for phosphorylation of Na_V_1.1 and Na_V_1.2 channels’ Ser residues in the Na_V_α-subunit’s intracellular loop, leading to the reduction in Na^+^ channel activity and conductance. High [Mg^2+^]*_i_* (10 mmol/L) can conversely lead to a decrease in PKA activity [72,76,77].

It is highly debatable whether high [Mg^2+^]*_i_* (30 mmol/L) elicits a negative shift in the Na_V_ channel’s voltage dependence due to cytoplasmatic membrane surface potential change [73], since there is a relatively low half maximal inhibitory [Mg^2+^]*_i_* (4 mmol/L) compared to high blocking [Mg^2+^]*_o_* (35 mmol/L) that reduces inward Na^+^ channel conductance [74].

##### Effects of Mg^2+^*_o_* on Na_V_ Channels

Electrophysiological examination of Mg^2+^*_o_* effects on murine hippocampal neurons presents a concentration-dependent increase in AP thresholds, resulting in decreased excitability. Higher [Mg^2+^]*_o_* (10 mmol/L) also reduces peak AP amplitude. As this action is comparable to the effect of tetrodotoxin (TTX), a highly selective antagonist of Na_V_ channels, it is assumed that an increase in [Mg^2+^]*_o_* leads to a decreased availability and activation of Na_V_ channels. Surface charge on the cell membrane created by sialic acid, phosphates, charged lipids, charged amino acids, and additional hydrophilic channel protein residues elicits local electrical fields near the channel voltage sensor [78]. However, some of the effects of Mg^2+^*_o_* on AP threshold and neuronal excitability are considered to result from surface charge screening, a process where extracellular cations bind to negative charges on the membrane, reduce surface potential, and produce local hyperpolarizing conditions for VSDs of Na^+^ channels. This charge screening effect is recognized as a depolarizing shift in Na^+^ voltage-dependent channel activation and inactivation [79]. Similar findings are seen in acutely isolated hippocampal CA1 neurons, as Mg^2+^*_o_* elicits a concentration-dependent reduction in Na_V_ channel conductance. Moreover, exposing the cells to a fixed [Mg^2+^]*_o_* at different holding membrane potentials elicits a progressive reduction in Na^+^ current amplitude, indicating the voltage-dependence of Mg^2+^*_o_*-induced channel block [80]. Na_V_ channel activity in murine hippocampal slices is susceptible to a concentration-dependent block by Mg^2+^*_o_*, as a decrease in Na_V_-mediated Na^+^ current amplitude is evident in high [Mg^2+^]*_o_*. On the other hand, exposing neurons to Mg^2+^*_o_*-free solution facilitates channel activation and leads to an increase in Na^+^ current amplitude [81]. In addition, studies on isolated murine saphenous nerves confirm that Mg^2+^*_o_* acts in a similar manner to TTX, via a concentration-dependent decrease in Na^+^ currents through Na_V_1.6 channels in Aβ fibers [82]. Altering the gating properties of the Na_V_ channel leads to AP threshold shift, spontaneous synaptic activity, ectopic neuronal charges, and epileptic activity in *in vitro* and *in vivo* models.

Depolarization of the resting membrane potential by Mg^2+^*_o_* followed by decreased excitability of neurons seems contradictory. To explain this effect, a relatively novel hypothesis suggests that Mg^2+^*_o_* may induce subthreshold depolarization by quantum tunneling through closed Na_V_1.2 channels. Namely, it was calculated that at a concentration of 5.5 mmol/L Mg^2+^*_o_* passes through the channel and its intracellular hydrophobic gate, by acquiring the kinetic energy from neuronal membrane voltage and the thermal body energy, thus eliciting minor membrane depolarization by 5 mV. Unlike Mg^2+^*_o_*, Mg^2+^*_i_* has a lower tunneling probability, since they have lower kinetic energy. The degree of neuronal membrane depolarization by Mg^2+^*_o_* is determined by the tunneling probability, Na^+^ channel density in the membrane, and [Mg^2+^]*_o_* [83].

#### 2.1.4. Mg^2+^ Effects on Large Conductance Ca^2+^-Activated Potassium (BK) Channels

BK channels have a large single-channel conductance. Activated by membrane voltage depolarization, micromolar [Ca^2+^]*_i_*, millimolar [Mg^2+^]*_i_*, or other ligands, BK channels mediate substantial K^+^ efflux and elicit repolarization of the membrane and/or closure of Ca_V_ channels to reduce Ca^2+^ influx. Assuming the role of a negative feedback to membrane depolarization and elevated [Ca^2+^]*_i_*, these channels regulate membrane excitability, intracellular ion homeostasis, and Ca^2+^ signaling, as they are often colocalized with Ca_V_ channels, N-methyl-D-aspartate receptors (NMDARs), and ryanodine receptors (RyRs) [84,85]. BK channels are expressed in skeletal and smooth muscles, neuron soma, axons, and presynaptic dendrite terminals, cochlear inner hair cells, and chromaffin cells, thus playing an important role in muscle contraction, neurotransmission, control of neuronal excitability (control of interspike interval and spike frequency adaptation), tuning hair cell firing, and hormone secretion. Native BK channels comprise α-subunits or a combination of α- and β-subunits (β1, β2/3, β4) or γ-subunits (γ1–γ4) in homotetrameric form [86,87]. Channels assembled from α-and β1-subunits are found in smooth muscle cells; ones made up of α-and β2/3-subunits exist in the brain, heart, and kidneys [86], while channels containing α-and β4-subunits are most abundant in the brain and spinal cord [87]. On the other hand, channels composed of α- and γ3-subunits are selectively expressed in the brain, while those built from α-and γ1-subunits are expressed in smooth muscle cells of cerebral arteries [87]. Each of the four α-subunits (Slo1 proteins) contains seven TM segments (S0–S6), including an additional S0 transmembrane segment that carries the N-terminus to the extracellular side and interacts with β-subunits, the pore–gate domain (PGD, S5–S6) with the C-linker (between S6 C-terminus and S7 N-terminus), which acts as an activation gate, and the voltage-sensing domain (VSD, S1–S4), whose positively charged Asp residues in the S4 helix represent a primary voltage sensor moving outward in response to membrane depolarization [88,89]. Furthermore, a large CTD or tail domain with four additional hydrophobic segments (S7–S10) is divided into two parts, homologous to a regulator of K^+^ conductance (RCK domains)–RCK1 (S7–S8) and RCK2 (S9–S10) [90] and serves as a primary ligand sensor [85]. RCK domains carry high-affinity Ca^2+^-binding sites–one on the RCK1 domain and one on the RCK2 domain (Ca^2+^ bowl), while the interface between the VSD and the cytoplasmatic domain and the RCK1 domain can be a target for Mg^2+^ binding [88]. BK channels owe their diversity to alternative splicing of *Slo1* mRNA and accessory β subunits, which modify pharmacology, voltage dependence, and channel kinetics [86,88]. The VSD senses voltage, and the cytoplasmatic domain senses intracellular ligands, and they both allosterically control K^+^ efflux through PGD in response to various stimuli, linking cellular metabolism and membrane excitability [84]. As Mg^2+^*_i_* is one of the ligands that modulates the activity of BK channels, we will mostly focus on its effects.

##### Effects of Mg^2+^*_i_* on BK Channels

Early experiments on planar lipid bilayers with incorporated murine muscle transverse tubule membrane [91,92] or parotid acinar cell membrane [93] fragments or on cultured hippocampal neurons [94] show that Mg^2+^*_i_* acts like an allosteric BK channel stimulator, increasing the channel open probability [91,92,93], the affinity of the channel for Ca^2+^*_i_* [91,92] and channel activation [93] in a concentration-dependent manner. Mg^2+^*_i_* is suggested to bind to channel’s modulatory sites on its cytoplasmic side distinct from Ca^2+^-binding sites, as either physiological [Mg^2+^]*_i_* (1 μmol/L to 1 mmol/L) or high [Mg^2+^]*_i_* (10 mmol/L) at constant [Ca^2+^]*_i_* increase in the cooperativity of channel Ca^2+^ activation, with a two-fold increase in the Hill coefficient for Ca^2+^_i_-induced channel activation curve [93]. Interestingly, in the absence of Ca^2+^*_i_*, Mg^2+^*_i_* fails to activate the channel [91,93,94], even at extremely high [Mg^2+^]*_i_* of 50 mmol/L [92]. Increasing [Mg^2+^]*_i_* when Ca^2+^*_i_* is significantly reduced recovers channel open probability and single-channel current amplitude, with Mg^2+^*_i_* achieving almost the same efficacy as Ca^2+^*_i_* [94]. These findings indicate that Mg^2+^*_i_* is a potential internal modulator of BK channels in Ca^2+^*_i_*-dependent regulation of neuronal excitability.

Electrophysiological studies on cultured murine skeletal muscle cells [95], cerebrovascular smooth muscle cells [96], rabbit pulmonary artery [97], portal vein and coronary artery [98] smooth muscle cells, phospholipid bilayers containing rabbit colonocyte membrane fragments [99] or *Xenopus* oocytes expressing wild-type [100,101,102,103,104] or mutant mouse Slo1 (mSlo1) BK channels [105] show that Mg^2+^*_i_* can decrease channel outward K^+^ current amplitude and conductance in a concentration-dependent and voltage-dependent manner, acting as a fast blocker. The magnitude of Mg^2+^*_i_*-induced blocking action can be attenuated by [K^+^]*_i_* increase [95,99]_._ Interestingly, certain millimolar [Mg^2+^]*_i_* can, however, enhance channel open probability independently of membrane potential [96,98,100,101]. Patch-clamp recordings of macroscopic currents on oocytes expressing mSlo1 channel corroborate earlier findings, but also demonstrate that, at positive membrane voltage, Mg^2+^*_i_* reduces outward current amplitude regardless of [Ca^2+^]*_i_*, while negative membrane potential relieves channels from Mg^2+^*_i_* block [100]. Furthermore, Mg^2+^*_i_* can block mutant channels lacking CTD Ca^2+^-binding sites, but it does not affect channel activation. It is also proposed that channel sites involved in Mg^2+^-dependent channel activation are distinct from the ones mediating the previously described Mg^2+^ block [100].

Mg^2+^*_i_* competes with Ca^2+^*_i_* for BK channel low-affinity metal-binding sites (Mg^2+^-or Mg^2+^/Ca^2+^-binding sites). This competition is more evident in the presence of millimolar [Ca^2+^]*_i_,* which, being higher than physiological levels by three orders of magnitude, activates channels not only by occupying their high-affinity Ca^2+^-binding sites, but also by competitive binding to low-affinity metal-binding ones [100]. Moreover, low-affinity binding sites exhibit greater affinity for millimolar [Ca^2+^]*_i_* than for millimolar [Mg^2+^]*_i_*, indicating that, at high concentrations, Ca^2+^*_i_* and Mg^2+^*_i_* can use these binding sites to modulate channel-gating kinetics [101]. In contrast, apart from binding to the Mg^2+^-binding site, which activates the channel, Mg^2+^*_i_* can bind to the high-affinity Ca^2+^-binding site, without any effect, competitively inhibiting Ca^2+^-dependent activation [100,102]. As [Ca^2+^]*_i_* increases, Mg^2+^*_i_* binding to high-affinity Ca^2+^-binding sites declines, but when [Mg^2+^]*_i_* reaches supraphysiological levels, this inhibition is preserved [100,102]. Further electrophysiological examination on *Xenopus* oocytes expressing mSlo1 channels demonstrates that millimolar [Mg^2+^]*_i_* added to saturation micromolar [Ca^2+^]*_i_* (~110 μmol/L) enhances Ca^2+^*_i_*-triggered channel activation by interacting with low-affinity Mg^2+^-binding site [101]. However, it is debatable whether Mg^2+^*_i_* can activate BK channels by binding to the high-affinity Ca^2+^-binding site and substituting Ca^2+^*_i,_* and if this activation can occur in Ca^2+^*_i_*-free conditions [91,92,93,94,100,101]. Since Mg^2+^*_i_* causes a shift in channel gating as a result of channel closure retardation, rather than fast channel opening, Mg^2+^*_i_* effects are considered to be dependent on the channel open conformation [100,101]. Activation of BK channels by Ca^2+^_i_ is probably potentiated by Mg^2+^*_i_* through channel allosteric modulation, as Mg^2+^*_i_* binding to low-affinity sites allosterically regulates channel open conformation, independently of Ca^2+^*_i_* binding to high-affinity binding sites and voltage sensor movement to membrane depolarization [101]. The binding of Ca^2+^*_i_* to the high-affinity binding sites, along with membrane depolarization, opens the channel, while Mg^2+^*_i_* bound to the low-affinity binding site potentiates channel activation.

In order to modulate BK channel activity, Mg^2+^*_i_* needs to simultaneously bind to several amino acid residues within the channel, prompting interdomain and intersubunit interactions. Studies on oocytes expressing mutant mSlo1 channel constructs reveal that the position 397 Gln oxygen-containing carbonyl group (Q397) coordinates Mg^2+^*_i_* ability to bind to Glu residues in 374 (E374) and 399 (E399) positions in the RCK1 domain [106,107]. Exchanging Gln with cysteine (Cys) in position 397 (Q397C) reduces the sensitivity of this mutant channel to Mg^2+^*_i_* both at zero and saturation [Ca^2+^]*_i_*, but it does not abolish it [106,107] and has no effect on the mutant channel conductance-voltage (G–V) relation [108]. Other mutant mSlo1 channel variants with Gln being replaced by arginine (Arg), Lys or tryptophan (Trp) (Q397R/K/W, respectively), exhibit decreased in Mg^2+^*_i_* sensitivity, but substitution with Glu (Q397E) or Asp (Q397D) enhances Mg^2+^*_i_* sensing, possibly due to the electrostatic attraction of negatively charged amino acid residues to bound Mg^2+^ [107,109] and shifts the channel G–V relation to more positive voltages [108]. Since Q397 is close to Mg^2+^*_i_*-binding sites E374 and E399, its mutation affects the channel’s Mg^2+^*_i_* sensitivity and binding affinity due to conformational changes in the binding sites or by an electrostatic interaction with already bound Mg^2+^ [107]. Interestingly, adding positive charge to this site, by substitution with Lys (Q397K) or Arg (Q397R) activates the mSlo1 mutant channel in absence of Mg^2+^*_i_* and shifts the G–V relation to more negative voltage, suggesting that the positively charged residue mimics Mg^2+^*_i_* bound to nearby sites (E374 and E399) and can electrostatically interact with positively charged Arg (R213) of the channel VSD [108]. Mutant Q397K or Q397R channels enhance the voltage sensor activation and mobility only when the channels are in the open state, similarly to wild-type channels upon Mg^2+^*_i_* binding [108]. Subsequent work on oocytes expressing several mutant channel variants reveals two additional Mg^2+^*_i_*-binding sites–a negatively charged, oxygen-bearing Asp residue in position 99 (D99) and a polar, uncharged Asn residue in position 172 (N172) in the α-subunit VSD, in addition to E374 and E399 in the RCK1 domain. Exchanging Asp in position 99 with nonpolar Ala (D99A) in the VSD makes the mutant channel not only resistant to Mg^2+^*_i_* but can abolish Mg^2+^*_i_* binding to E374 and E399 sites in the RCK1 domain. Furthermore, mutant channels where Asp is substituted by amino acids lacking oxygen in their side chain, such as Cys, Trp, Arg, or Lys (D99C/W/R/K, respectively), lose Mg^2+^ sensing as well. Conversely, Mg^2+^*_i_* can still exert its activating effect on a channel with preserved side chain oxygen, namely when Asp is interchanged with Gln, Asn, or Glu (D99Q/N/E, respectively) [109]. BK channels with a mutation of the other binding site, namely Asn replacement with Ala in position 172 (N172A), show a substantial decline of channel responsiveness to Mg^2+^*_i_*, whereas substitution with Arg (N172R) or Lys (N172K) abolishes Mg^2+^ sensitivity of the channel. Having a negatively charged Glu or Asp residue instead of Asn (N172D or N172E, respectively) enhances the mutant’s binding site interaction with Mg^2+^*_i_* [109]. Double mutant channels bearing Cys instead of Asp in position 99 and Gln in position 397 (D99C:Q397C) show that the VSD and RCK1 domains are close enough that a disulfide bond forms between their cysteine residues. This proves that the spatial proximity between D99 in VSD and E374 and E399 in the RCK1 domain fits the dimensions of a Mg^2+^-binding site [109]. Furthermore, double mutant channels comprising N172D substitution with either D99A, E374A, or E399N exhibit considerably stronger sensitivity to Mg^2+^*_i_* than those carrying single mutations, indicating that the Asp carboxylate group oxygen in position 172 rescues the channel’s ability to bind Mg^2+^*_i_* [109]. Experimental findings propose that Mg^2+^*_i_* binds to a complex site that consists of Asp residues at position 99 (D99) and Asn residues in position 172 (N127) in the VSD of one α-subunit and Glu residues at positions 374 (E374) and 399 (E399) in the RCK1 domain of a neighboring α-subunit, mediating an intersubunit interaction between the voltage-sensing and ligand-sensing domains [109]. This allows for the activation of the channel by electrostatic interaction between bound Mg^2+^*_i_* and the positively charged Arg (R213) residue of the VSD in the S4 segment [109]. Mutant mSlo1 channels with only preserved low-affinity Mg^2+^-binding site open to an increasing membrane voltage after a markedly shorter latency period in the presence of Mg^2+^*_i_* and a decrease in the interval duration of the channel closed state. Furthermore, Mg^2+^*_i_* prolongs bursts of channel openings with a higher channel opening rate, while the mean open interval duration increases in 2-fold compared to zero [Mg^2+^]*_i_* conditions. Mg^2+^*_i_* can bind to closed channels, while increasing the probability of channel opening, but is more efficient in binding to open channels, decreasing the probability of channel closing and closing rates [105].

Movement of the voltage sensor in response to membrane voltage change [108,110] generates a transient gating current (*I_g_*) [108]. Whereas having no effect on the *I_g_*_ON_, caused by voltage sensor movement from the resting to the activated state, when most channels are closed, Mg^2+^*_i_* slows down the return of the voltage sensor from the activated to the resting state, when most channels are open, and decreases the amplitude of the generated *I_g_*_OFF_ [108]. Mutant channels where Asp is exchanged with Ala in position 99 (D99A) or position Glu is replaced with Asn in position 399 (E399N) become resilient to Mg^2+^*_i_* effect on *I_g_*_OFF_ [109]. The transmembrane S4 segment is not only the primary voltage sensor that leads to the opening of the channel, but also the C-linker activation gate [110] but also mediates Mg^2+^*_i_*-dependent channel activation cascade. Channels carrying mutations of the VSD in the S4 segment, namely position 213 Arg substitution with polar uncharged Gln (R213Q) [110] or Cys (R213C) [108] have altered voltage dependence and lose sensitivity to Mg^2+^*_i_* and Mg^2+^*_i_*-dependent activation [108,110]. Time course of wild-type channel deactivation in the presence of Mg^2+^*_i_* is slower than in zero [Mg^2+^]*_i_*, but the R213Q mutant becomes unaffected by changes in [Mg^2+^]*_i_* [110]. It is shown that Mg^2+^*_i_* bound to E374 and E399 in the cytoplasmatic RCK1 domain can come close to the VSD in the S4 segment and electrostatically interact with the VSD positively charged R213 site, which is important for voltage sensing. The created electrical field at R213 and the repulsion of positive charges promote activation of the voltage sensor and favor the channel open state. It is proposed that VSD senses not only membrane voltage but also the bound Mg^2+^*_i_*. Therefore, the R213 residue acts both as a membrane voltage sensor and Mg^2+^*_i_* binding sensor, so that VSD can control channel activation [108]. Excitatory Mg^2+^*_i_* effect on BK channels requires activation of the channel voltage sensor. Substitution of Arg in position 210 with Cys leads to a constitutively active voltage sensor in the mutant channel, which can be opened by Mg^2+^*_i_* regardless of the membrane voltage. In addition, the channel’s open probability increases with increasing [Mg^2+^]*_i_* when they are activated [104,105]. However, it is possible that high [Mg^2+^]*_i_* (10–100 mmol/L) elicits a direct effect on channel opening independently of voltage sensor activation, similarly to that of Ca^2+^*_i_*, through an additional low-affinity binding site [104]. Mg^2+^*_i_* strengthens the coupling of voltage sensor activation and channel opening by interacting with multiple sites on the voltage sensor [104].

BK channel reactivity to Mg^2+^*_i_* is also affected by α-subunit mutations in the C-terminal half of the S4 segment and the N-terminal part of the S4–S5 linker. Specifically, mutant channels where Gln in position 216 or 222 is replaced with Arg (Q216R or Q222R, respectively), Glu in position 219 substituted with Gln (E219Q), leucine (Leu) in position 224 exchanged with Arg (L224Q) or channels bearing double mutation at positions 219 and 222 (E219R/Q222R) have a suppressed sensitivity to Mg^2+^*_i_*, albeit some of the positions in wild type channels do not directly participate in Mg^2+^*_i_* binding. These findings suggest that interactions between amino acid residues in the C-terminal part of S4 and the N-terminal part of S4–S5 linker with adjacent voltage-sensing moieties are crucial for Mg^2+^*_i_*-dependent channel activation [110]. Moreover, the interaction between the RCK1 domain and the voltage sensor conveys the energy from Mg^2+^*_i_* binding to open the channel gate [110]. Electrophysiological experiments on oocytes expressing wild-type mSlo1 channels propose that a ring composed of eight negatively charged Glu residues in positions 321 and 324 at the cytosolic channel entrance might be a possible target for Mg^2+^*_i_* blocking action. Moreover, mutant channels without the ring structure, stemming from Glu substitution with polar uncharged Asn (E321N:E324N), exhibit a significant resistance to Mg^2+^*_i_*-induced blocking action [103]. Substantial [K^+^]*_i_* increase equally mitigates Mg^2+^*_i_* block of wild type and mutant channels, suggesting that, when present, the ring of negative charge cannot facilitate blocking action in high [K^+^]*_i_* as it loses electrostatic attraction for Mg^2+^*_i_* and that there might be an additional site of Mg^2+^*_i_* action as the block is not abolished [103]. Namely, not only is Mg^2+^*_i_* attracted to the inner opening of the BK channel, making it easier to block K^+^ currents, but the screening of the negative charge by Mg^2+^*_i_* decreases in the polar attraction of the channel opening for cytosolic K^+^ and the outward current [103].

##### Effects of Mg^2+^*_o_* on BK Channels

Studies examining the effects of [Mg^2+^]*_o_* change on BK channel activity are scarce, as most studies investigate Mg^2+^*_i_*. Earlier electrophysiological experiments on murine muscle transverse tubule membranes incorporated into planar lipid bilayers indicate that high [Mg^2+^]*_o_* exerts no effect on BK channel activation or channel affinity to Ca^2+^ [91,92]. Subsequent studies on colonocyte membrane fragments incorporated into phospholipid bilayers; however, show that Mg^2+^*_o_* decreases in the channel outward current amplitude and channel conductance altogether in a concentration-dependent manner, suggesting that this might be a result of electrostatic screening of negative charges located at the channel’s extracellular pore opening [99,111].

#### 2.1.5. Mg^2+^ Effects on Small Conductance Ca^2+^-Activated Potassium (SK) Channels

The SK channels are responsible for the medium membrane afterhyperpolarization (AHP) following APs, thus modulating intrinsic neuronal excitability and controlling spike firing rates and AP frequency, regulating dendritic excitability [112]. These channels are built as homotetramers of α subunits composed of six TM segments (S1–S6), with the PGD (S5–S6) surrounded by S1–S4 TM segments. Unlike BK channels, SK channels are voltage-insensitive and activate in response to low [Ca^2+^]*_i_* interacting with their CaM-binding domain (CaMBD). Each subunit binds one CaM through an interaction between the CaM C-lobe with the CaMBD independently of Ca^2+^*_i_* and between the CaM N-lobe and S4–S5 linker. In response to Ca^2+^*_i_*, the S4–S5 linker elicits a set of conformational changes, causing the S6 TM segment to move and open the channel pore for K^+^ efflux. SK channels are primarily expressed in the CNS, PNS, and the cardiovascular system [89]. Activation of SK channels in *substantia nigra*, ventral tegmental area, and cerebellar dopaminergic (DA) neurons to counteract Ca_V_ channel-or NMDAR-mediated Ca^2+^ influx and membrane excitation regulates muscle coordination and movement [113,114]. SK1 channels are expressed in the neocortex and co-expressed with SK2 channels in the hippocampus, thalamus, cerebellum, and brain stem, while SK3 channels are expressed in the midbrain and the cerebellum [89]. They are usually activated by Ca^2+^ influx through Ca_V_ channels during AP. SK channels couple their activity with postsynaptic modulators of [Ca^2+^]*_i_*, like NMDARs, in dendritic spines of neurons in the hippocampus and amygdala. They are involved in the regulation of the excitatory postsynaptic potential and can influence NMDAR activation through voltage-dependent Mg^2+^ block, affecting synaptic plasticity [115]. Inhibition of dendritic SK channels leads to long-term potentiation (LTP) and has an important role in memory and learning [116,117]. In cardiac atria and ventricles, SK channels act as an important negative feedback mechanism to [Ca^2+^]*_i_* increase through Ca_V_1 channel activation or Ca^2+^ release from cellular storage, and lead to repolarization of cardiomyocytes and AP completion [118]. Interestingly, the evidence of direct Mg^2+^*_i_* effect on the function and gating kinetics of SK channels is scarce, while there are still no studies showing the effects of Mg^2+^*_o_*.

Patch-clamp recordings of *Xenopus* oocytes expressing cloned rat SK2 (rSK2) channels [119,120] or freshly isolated murine aorta endothelial cells [121] show that increasing [Mg^2+^]*_i_* within the physiological range reduces outward K^+^ channel currents [119,120,121]. Supraphysiological [Mg^2+^]*_i_* (up to 10 mmol/L) blocks the channel completely. Depolarizing shifts in membrane voltage enhance Mg^2+^*_i_* blocking action as if Mg^2+^*_i_* are pushed into the channel. Furthermore, increasing [Mg^2+^]*_i_* leads to inward rectification of the channel current-voltage (I–V) relation in a voltage-dependent manner [119,121]. Conversely, SK channel block elicited by high [Mg^2+^]*_i_* is attenuated with increasing [Ca^2+^]*_i_*, while a decrease of [K^+^]*_o_* potentiates the blocking effect and reduces its voltage-dependency [119]. Explaining the mechanisms of SK channel behavior in these conditions leads to the conclusion that they act similarly to Kir channels, so they can be referred to as “Ca^2+^-activated inward rectifier K^+^ channels” [119]. Electrophysiological studies on oocytes expressing mutant rSK2 channels were used to examine possible metal-binding sites in the channel pore-forming region that can mediate the Mg2+-induced block [120]. Mutant rSK2 channels where Ala substitutes Trp in position 379 (T379A), 398 (T398A), or Cys in position 386 (C386A) exhibit a comparable degree of channel block by divalent cations to that of wild-type channels. Conversely, replacement of Ser in position 359 with Ala (S359A) decreases the mutant channel susceptibility to any blocking action, proving that this is the intended site for direct interaction with Mg2+, among other divalent cations, and that it determines channel ionic selectivity. Mg^2+^*_i_* binding affinity to the S359A mutant rSK2 channel increases at lower positive membrane voltages [120]. Knowing that the activity of these channels is coupled with Ca^2+^*_i_* balance modulated by Ca_V_ channel, NMDAR, and nAChR activity, it is plausible that Mg^2+^*_i_* and Mg^2+^*_o_* can also indirectly affect SK channel gating kinetics by altering [Ca^2+^]*_i_*.

### 2.2. Ligand-Gated Ion Channels Regulated by Mg^2+^

Ligand-gated ion channels are oligomeric protein structures that convert a chemical signal into an ionic current flux through the central pore of the channel. They are involved in crucial signal transduction in the CNS and PNS. The canonical activation pathway of these channels starts with chemical messenger activation, i.e., ligand binding, conformational change, and restructuring of the channel’s integral protein components, opening of the channel pore, and ion flux across the membrane that alters the cell’s membrane potential and ends with a cellular response. The channel pore is permeable to cations such as Na^+^, K^+,^ and Ca^2+^ or anions such as Cl^−^, with certain selectivity determined by ion size and charge. Here we present the findings concerning Mg^2+^ effects on several ligand-gated ion channels of ionotropic receptors, cationic channels of the N-methyl-D-aspartate (NMDA) glutamatergic receptor and purinergic P2X receptors, Cl^−^ ion channel of type A receptor for gamma-Amino Butyric Acid (GABA_A_R), and cationic channel of the nicotinic cholinergic receptors (nAChR). There are a few studies demonstrating the effects Mg^2+^ exerts on GABA_A_R and nAChR function. We present their findings in two short overviews.

#### 2.2.1. Mg^2+^ Effects on N-Methyl-D-aspartate Receptors (NMDARs)

The NMDARs belong to a family of ionotropic glutamate receptors (iGluRs) known for their essential contribution to excitatory synaptic plasticity and long-term signaling in the CNS. These are heterotetrametric structures composed of two GluN1 subunits and two of four types (A–D) of GluN2 subunits or two types (A–B) of GluN3 subunits. Each receptor subunit contains an extracellular amino-terminal domain (ATD), an extracellular agonist-or ligand-binding domain (LBD), three TM regions (TM1, TM3, and TM4), a re-entrant p-loop (M2 region) with a pore-lining segment and a selectivity filter, and an intracellular C-terminal domain [122,123]. Mg^2+^ plays a pivotal role in modulating NMDAR activity. Activation of the NMDAR is unique as it requires two signaling factors–Gly binding to a site on the GluN1 subunit and the agonist (NMDA, Glu, Asp) binding to a site on the GluN2 subunit in coordination with depolarization-dependent removal of Mg^2+^ from its binding site on the GluN2 subunit. Most excitatory synapses function as a result of AMPA (α-amino3-hydroxy-5-methyl-4-isoxazole propionic acid) and NMDA receptor interplay, and the resulting excitatory postsynaptic currents (EPSC) reflect this heterogeneity. Upon membrane voltage change stimulus, AMPA receptor (AMPAR) currents rise and subside fast, as they determine the onset and maximal amplitude of the EPSC, while NMDAR currents rise and decline more slowly, setting the decay of the EPSC and strongly influencing total positive charge entering the cell [124].

##### Effects of Mg^2+^*_i_* on NMDARs

Mg^2+^*_i_* can block NMDAR channels, and this blocking effect has yet to be explained for its physiological function. Electrophysiological examination of inside-out patches from oocytes expressing NMDAR GluN1 and GluN2A subunits shows that increasing [Mg^2+^]*_i_* in Ca^2+^*_o_*-free solution at positive membrane potentials reduces outward currents through the NMDAR channel. However, at more negative membrane potentials, blocking of inwardly directed currents by Mg^2+^*_i_* is stronger [125]. Mg^2+^*_i_* produces only slight inhibition of inward currents in CA1 neurons at negative potentials but has substantial blocking activity on outward currents at positive membrane potentials. The blocking effect of Mg^2+^*_i_* is more pronounced in conditions lacking Mg^2+^*_o_* [126]. Experiments on outside-out patches of neuronal membranes show that physiological [Mg^2+^]*_i_* can rapidly block the NMDA-activated channel, as it reduces the channel’s current amplitude at positive membrane potentials, without any flickering. This effect, which is augmented with increasing [Mg^2+^]*_i_*, is reversible and not detectable at negative membrane potentials [127,128,129]. The absence of the flickering activity during electrophysiological recording of cultured murine cortical neurons was hypothesized to be due to Mg^2+^*_i_* association and dissociation rates that are so high that the discrete open and blocked states cannot be discerned with signal filtering. The blocking rate constants increase with rising [Mg^2+^]*_i_* and membrane depolarization [129].

In contrast to Mg^2+^*_o_* eliciting channel blocking action by binding to Asn residues of GluN2 subunit, the blocking action of Mg^2+^*_i_* is primarily the result of an interaction with Asn residues in the channel’s GluN1 subunit. Mutant NMDAR expressed on oocytes where GluN1 subunit’s Asn is position 598 is exchanged with nonpolar Gly (N598G) or polar uncharged Gln (N598Q) or Ser (N598S) attenuates the Mg^2+^*_i_*-elicited channel block [125,130,131], while substitution with Asp (N598D) enhances the blocking action due to the negatively charged Asp residue. Asn residues of both GluN subunits form a narrow constriction of the channel pore and represent binding sites for Mg^2+^*_i_*, although GluN1 is the dominant binding site [125,131].

In addition to the binding site for Mg^2+^*_o_* and blockage deep in the pore (~0.64 through the electric field of the membrane from the extracellular side) [127,132], NMDAR channel possesses a divalent cation binding site near the external mouth of the pore (~0.2 through the electric field), to which Mg^2+^ binds slowly [133]. The difference in the channel’s blocking activity by Mg^2+^*_i_* and Mg^2+^*_o_* [127,130], as well as the markedly faster unblocking rate constants of Mg^2+^*_i_* at physiological voltage [128], suggest that Mg^2+^*_i_* and Mg^2+^*_o_* bind at different sites within the channel pore.

On the other hand, it is proposed that Mg^2+^ can unblock the deep binding site of the NMDAR channel either by moving back to the extracellular solution or by permeating the channel to the intracellular compartment [134]. Assuming that there are multiple binding sites in a narrow NMDAR channel pore, any other ions binding to more external sites than the one Mg^2+^ already binds to will trap Mg^2+^ in the pore (“lock-in effect”), enhancing the channel block [132,134,135]. Furthermore, some other cations from extra- and intracellular fluid, such as Ca^2+^*_o_* [133], Na^+^*_i_* or Na^+^*_o_* [132,134,136] or Cs^+^*_i_* [136], when occupying more external permeant binding sites of the channel pore, prevent Mg^2+^*_o_* from binding to their deep blocking site. Mg^2+^ can still occupy the blocking site in the NMDAR channel pore while the receptor agonist unbinds from its site, which is considered to be a trapping-block kinetic scheme. What is more interesting is that the closing rate constant of the blocked channel is several times faster than that of an unblocked channel, leading to an asymmetrical trapping-block kinetic scheme. This model suggests a link between Mg^2+^ presence in the NMDAR channel pore and its allosteric influence on the permeation gate closure [137].

##### Effects of Mg^2+^*_o_* on NMDARs

Mg^2+^*_o_* are well-known voltage-dependent blockers of NMDARs. Earlier works on murine neuron cultures [138,139] or neurons in murine hypothalamic slices [140] show that NMDAR response to Glu is potentiated when [Mg^2+^]*_o_* is reduced to subphysiological levels (below 1 mmol/L). It was then proposed that the voltage-dependence of NMDAR conductance is a result of a voltage-dependent Mg^2+^*_o_* block, as Mg^2+^ enters the receptor-channel, sensing the membrane electric field. Moreover, Mg^2+^*_o_*-free solution allows Glu, Asp, or NMDA to elicit stable inward currents, almost linearly with increasing membrane potential [138,139,140], but addition of Mg^2+^*_o_* strikingly reduces inward currents, while causing little change to outward currents [138,139]. Glutamate or NMDA in Mg^2+^*_o_*-free conditions is capable of opening the NMDAR cationic channel independently of the membrane potential level, showing reduced voltage sensitivity of the channel conductance in the absence of Mg^2+^*_o_* [138,139,140]. Elevated [Mg^2+^]*_o_* in isolated murine hippocampal neurons can potentiate outward currents through NMDAR channels in a concentration-dependent manner [141]. Under Mg^2+^*_o_*-free conditions, NMDA-induced nerve cell membrane depolarization in murine hippocampal slices [142], cerebral cortex slices [143,144] or spinal cord culture [139] is accompanied by a decrease in membrane input resistance. Conversely, [Mg^2+^]*_o_* increase elicits a persistent rise in the input resistance due to a voltage-dependent Mg^2+^ block of NMDA-evoked current [139,142,143,144]. NMDAR response to NMDA seen in neurons of murine brain slices or cultures at physiological [Mg^2+^]*_o_* (~1 mmol/L) exhibits a negative slope in the I–V relation, i.e., a conduction decrease over the membrane potential range of −70 to −30 mV, an effect that is absent in Mg^2+^*_o_*-free solution or at more positive membrane potentials and depolarization [139,140,141,145,146]. Hyperpolarization of the cell membrane potentiates the blocking effect of Mg^2+^*_o_* [138], but that effect subsides at positive membrane potentials (+20 mV) [145].

Morphological studies of neurons in culture report on the toxic effects of NMDA or Glu in Mg^2+^*_o_*-free conditions, suggesting that the relief from Mg^2+^*_o_*-induced block of NMDA-activated channels drives a more persistent stimulation of the cell membrane and influx of Ca^2+^ ions [147,148,149]. When membrane patches of neurons in brain slices or culture are held at negative membrane potential in the presence of Mg^2+^*_o_*, each stimulated channel opens in a form of grouped bursts, a flickering activity whose duration decreases as [Mg^2+^]*_o_* increases [135,138,139,143,150,151]. These bursts are a result of short current flow interruptions during periods when the channel is in the open configuration [151], implying a possibility that Mg^2+^*_o_* enters and blocks the open channel very briefly at a time [150]. In addition, Mg^2+^*_o_* reduces the frequency of the Glu-induced NMDAR channel open state [138]. The NMDAR channel sensitivity to Mg^2+^*_o_* block is suggested to result from Asn residues in the pore-forming TM2 segment acting as potential binding sites for Mg^2+^*_o_*. Electrophysiological experiments on mutant channels expressed on *Xenopus* oocytes demonstrate that the replacement of Asn with Gln in position 598 (N598Q) of the GluN1 subunit’s TM2 segment slightly reduces the Mg^2+^*_o_* block and decreases channel pore permeability for Ca^2+^ [152,153]. The same change in homologous position 595 (N595Q) of the GluN2 subunit (known as the Asn, N-site or Gln/Asn/Arg, Q/N/R-site) strongly diminishes Mg^2+^*_o_* blocking action and promotes Mg^2+^ ion permeability [152]. In the narrow constriction of the NMDAR channel, two adjacent GluN2 subunits align so that their Asn residues at positions N and N + 1, respectively, form a critical blocking site for Mg^2+^*_o_* [125]. However, GluN2 subunit’s differences in susceptibility to Mg^2+^_o_ block cannot be explained by a single structural determinant such as Asn at the N-site. Namely, chimeric GluN2 subunits in channels expressed on *Xenopus* oocytes, constructed by replacing portions of the least sensitive GluN2C subunit with elements from the most susceptible GluN2B subunit, emphasize three determinants–the TM1 segment, the linker between TM2 and TM3 segments, and the TM4 segment as being important for Mg^2+^-dependent block in addition to the Q/N/R-site [154]. Large rapid membrane depolarizations consequently affect NMDAR channels (comprising GluN1 and GluN2A or GluN2B), by alleviating Mg^2+^*_o_*-imposed block with a complex time dependence [137,155]. The resulting release of Mg^2+^*_o_* block and elicited current has a relatively fast component due to the rapid Mg^2+^ unbinding kinetics (time constant 1 ms or less), and a slow component from an inherent voltage-dependent channel gating which increases in its open probability (time constant 10–15 ms) [137,156]. It is suggested that the specific slow Mg^2+^*_o_* unblock and the voltage-dependent gating properties of NMDAR GluN2 subunits are attributed to the Ser residue at the 632 position (S632) in the TM3 region of GluN2 subunits, as substitution of Ser with Leu at this site (S632L) reduces the subunit’s affinity to Mg^2+^ [156,157]. Both Mg^2+^*_o_* and Gly are necessary for the physiological function of NMDARs. Interestingly, Mg^2+^*_o_* allosterically increases NMDAR affinity for Gly, and it reduces the desensitization of the receptor by interacting with the Gly binding site [141].

In more recent studies on murine CA1 neurons, it is suspected that the blocking action of Mg^2+^*_o_* is possibly not solely regulated by changing membrane potential, and that a relative proportion of Na^+^ influx and efflux is an important factor. Changing [Na^+^]*_i_* and [Na^+^]*_o_* to create an inward or outward Na^+^ ion flux alters the efficacy of the Mg^2+^ block. Presence of Mg^2+^*_o_* elicits a prominent current inhibition during Na^+^ inward flux but has little blocking effect when Na^+^ outward flux occurs. The flow and tendency of movement of permeant ions such as Na^+^ can enhance the Mg^2+^*_o_*-induced block of the NMDAR channel, rather than interfering with it [126].

#### 2.2.2. Mg^2+^ Effects on Purinergic P2X Receptors (P2XRs)

Purinergic P2 receptors are membrane proteins activated by extracellular nucleotides, which are widely expressed in many tissues and mediate cell communication. P2X receptors are ligand-gated channels conducting cation currents, while P2Y are G-protein coupled receptors. P2X (seven subtypes, P2X1-P2X7) mediate rapid cellular responses by opening their ligand-gated nonspecific cation channels in the cell membrane in response to ATP binding. These are unique, trimeric ATP-gated ion channels distinct from other transmitter-gated channels, forming pores that nonselectively allow small positive ions of Na^+^, K^+,^ and Ca^2+^ to flow through. Most of the established Mg^2+^ actions on P2XRs arise from outside the cell, through changes in ATP speciation and potential allosteric modulation, while direct, defined intracellular mechanisms are less well characterized.

##### Effects of Mg^2+^*_i_* on P2XRs

P2XRs possess intracellular N- and C-termini and cytoplasmic domains essential for receptor function and downstream signaling. Their role is currently emphasized in channel gating rather than specific modulation by Mg^2+^*_i_*. Given the nonselective cation permeability of P2XR channels, interaction with Mg^2+^*_i_* could, in principle, influence their permeation or rectification. However, current data do not assign a direct effect of Mg^2+^*_i_* on P2X receptors, as no defined specific, reproducible Mg^2+^*_i_* binding sites or block phenomena have been revealed as canonical modulators across P2XR subtypes [158]. Interpretations of Mg^2+^ effects rather center on extracellular ATP/Mg-ATP dynamics [159].

##### Effects of Mg^2+^*_o_* on P2XRs

Available structural and pharmacological data primarily detail extracellular P2X control by Mg^2+^*_o_*. Namely, Mg^2+^*_o_* profoundly shapes P2XR activation by altering the chemical form of ATP and, in some cases, by interacting with allosteric sites on the ectodomain. Under physiological conditions, ATP is predominantly complexed with Mg2+, as it neutralizes the negative charges on its phosphate groups and stabilizes the molecule conformation into a compact Mg–ATP complex. P2X subtype-specific activation is associated with ATP speciation in such a way that the active nucleotide form differs by receptor subtype: P2X2 can be robustly activated by free ATP (ATP^4−^), whereas Mg-ATP^2−^ promotes opening with very low efficacy at P2X2Rs, revealing a striking dependence of gating on the ATP speciation. Other subtypes also exhibit distinct profiles, establishing subtype-specific control by Mg^2+^*_o_* via the free ATP/Mg-ATP balance [160].

Human P2X2R subtype shows Mg^2+^*_o_*-dependent reduction in activation (while ligand binding remains relatively intact), P2X3R exhibits decreased activation at high Mg^2+^*_o_* levels with increased binding, while P2X2/3 heteromers display a hybrid effect, as determined using fluorescent ATP derivatives. These data indicate that Mg^2+^*_o_* causes P2XR binding–gating dissociation, as it can reduce gating efficacy without proportionally diminishing orthosteric binding, consistent with Mg-ATP favoring binding yet being a weaker gating agonist in certain subtypes. Additionally, evidence from ligand-binding and activation studies supports putative allosteric Mg^2+^*_o_* interactions, whereby Mg^2+^*_o_* can act beyond orthosteric Mg-ATP formation, potentially engaging an extracellular allosteric site to modulate P2XR function. This has been proposed to explain Mg^2+^*_o_* -dependent shifts in activation independent of binding loss, with subtype variability in magnitude and direction of the effect [161]. There is now an emerging recognition from subtype-specific experiments that Mg^2+^*_o_* is a functionally relevant P2XR modulator [162].

#### 2.2.3. Mg^2+^ Effects on Gamma-Amino Butyric Acid A Type (GABA_A_) Receptor

GABA is one of the most important inhibitory neurotransmitters in the CNS, and most of its actions are mediated by GABA_A_Rs. These receptors consist of subunits that belong to seven classes, and each class has one or several members–α (1–6), β (1–4), γ (1–4), δ1, ε1, π1, or ϑ1. Being usually pentameric, GABA_A_Rs are a result of combining two α, two β, and one γ, δ, ε, π, or ϑ subunit (2:2:1 ratio) [163].

Electrophysiological experiments on *Xenopus* oocytes, expressing recombinant GABA_A_Rs (α1β2γ2S, α2β2γ2S, α1β2, and α2β2), show a biphasic change in GABA-induced inward Cl^−^ currents in the presence of Mg^2+^*_o_*. An increase in inward current elicited by GABA binding to its receptor is evident in low [Mg^2+^]*_o_* (0.01 mmol/L), leading to a peak increase when the extracellular solution contains physiological [Mg^2+^]*_o_* of 1 mmol/L. However, high [Mg^2+^]*_o_* (10 mmol/L) reduces the GABA-activated current in most receptors [164]. Autoradiographic analysis of murine brain sections points to a decrease in the binding of a GABA_A_R Cl^−^ channel blocker (t-butylbicyclophosphoro[S^35^]thionate, [S^35^]TBPS) across various anatomical structures in the presence of physiological [Mg^2+^]*_o_* (1 mmol/L), an effect that achieves reversed by high [Mg^2+^]*_o_* reaching supraphysiological level of 10 mmol/L. Furthermore, Mg^2+^*_o_* potentiates GABA-induced inhibition of Cl^−^ channel blocker binding but also decreases the binding action of Cl^−^ channel blocker in the presence of a GABA_A_R competitive antagonist (gabazine, SR-95531). It is suggested that Mg^2+^*_o_* exerts action on GABA_A_Rs independently of GABA presence [164]. Effects of Mg^2+^*_o_* on GABA_A_Rs are potentially mediated by two receptor binding site types–a high-affinity potentiating site and a low-affinity inhibitory site.

#### 2.2.4. Mg^2+^ Effects on Nicotinic Acetylcholine Receptors (nAChRs)

The nAChRs are concentrated at the vertebrate neuromuscular junction, at synapses on autonomic ganglion cells and central neurons. nAChR is a pentameric structure, where each of the five subunits is arranged around the central pore. They can be homomeric, where subunits are identical, or heteromeric, where different subunits complex together. Neuronal nAChRs are heteromeric α4β2 pentamers formed in a 2:3 or 3:2 subunit ratio, while muscle nAChRs are most commonly composed of two α1- and one of β1-, δ-, and γ-or ε-subunits. Each subunit has a long extracellular N-terminal domain acting as an ACh- or agonist-binding site (extracellular domain, ECD), four TM regions (TM1–4), an intracellular loop that connects TM3 and TM4, and a short extracellular C-terminal domain. The TM2 regions of all five subunits form the lining of the receptor-associated ion channel pore [165].

The mechanism of nAChR activation by ACh has been examined. It proposes that two agonist acetylcholine oxyanions (ACh^+^) react with the nAChR recognition site and exchange for one Mg^2+^ ion. This exchange with Mg^2+^ occurs at two closely positioned negatively charged groups within the nAChR recognition site. The resting state of the membrane potential allows for electrostatic attraction between these negatively charged groups and Mg^2+^. However, when Mg^2+^ is replaced with two acetylcholine (ACh) molecules, they form two mutually repelling ACh^+^ receptor dipoles that cause the receptor groups to be forced apart, opening the receptor pore [166].

Mg^2+^*_o_* alters the time course of synaptic currents through nAChR channel, as excitatory miniature end-plate currents elicited by ACh binding to this receptor, exhibit slower decay when [Mg^2+^]*_o_* is higher [167]. Since receptor’s channel kinetics and channel gating determine current dynamics, two mechanisms have been proposed–cations such as Mg^2+^*_o_* interact with binding sites within the channel pore, and they alter the surface potential near the nAChR, indirectly influencing channel gating properties [168,169].

Patch-clamp recordings of sympathetic ganglion neurons and PC12 cells show that Mg^2+^*_i_* can block the channel pore, and that this blocking action is potentiated by membrane depolarization, i.e., it is voltage-dependent. However, extremely positive membrane potentials elicit permeation of the channel by Mg^2+^ and an outward current [170,171]. Electrophysiological examination of single-channel currents from cell-attached and outside-out patches of PC12 cells [169,171,172], guinea pig outer hair cells [173] or *Xenopus* oocytes expressing nAChRs [174] demonstrate that channel’s outward conductance is more noticeably reduced in the presence of high [Mg^2+^]*_i_*, an effect that is reversed when the intracellular solution dialyzing the cells is depleted of Mg^2+^*_i_* [169,171,172,173,174]. It is suggested that Mg^2+^*_i_* blocks the channel pore and changes the intrinsic channel gating of nAChR channel, and that both of these processes are voltage-dependent [169]. High [Mg^2+^]*_o_* decreases in ACh-evoked single-channel inward conductance, while conductance increases in Mg^2+^*_o_*-free solutions [171,172,173].

On the other hand, the effects of Mg^2+^*_o_* on outward conductance and Mg^2+^*_i_* on inward conductance of nAChR channels are debatable, as the currents are either not being affected [172] or being reduced [171]. Additionally, inward nAChR channel conductance is either not affected by varying [Mg^2+^]*_i_* [172], or slightly decreased with elevated [Mg^2+^]*_i_* [171]. The most likely explanation for the effects of both Mg^2+^*_i_* and Mg^2+^*_o_* on nAChR channel conductance is the screening of negative charges on both ends of the channel pore [172]. nAChRs are more sensitive to [Mg^2+^]*_i_* increase, blocking the outward currents, than to increasing [Mg^2+^]*_o_* blocking the inward currents, showing the asymmetrical effect of Mg^2+^ on nAChRs [171,172].

**Figure 2:**
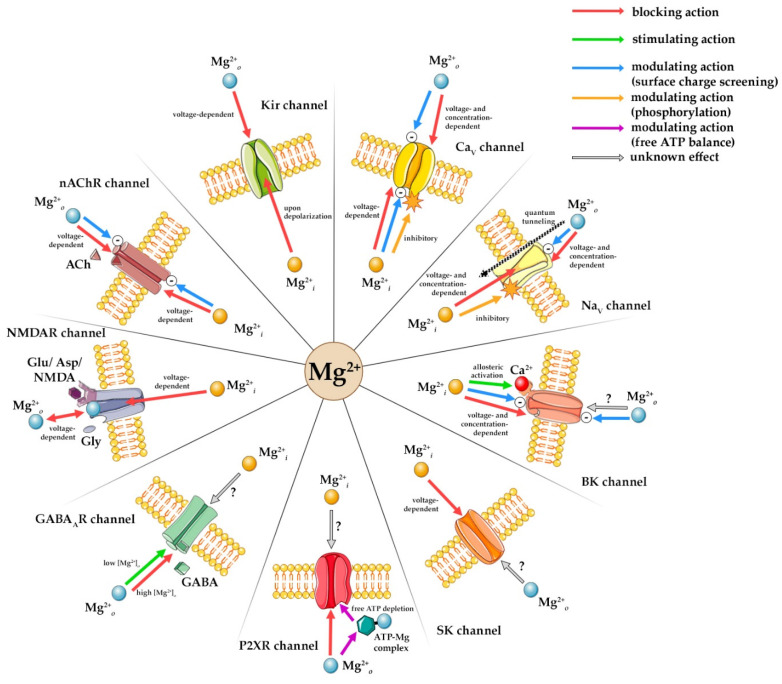
Magnesium ions as modulators of ion channel function in the neuronal membrane. Mg^2+^*_i_*—intracellular Mg^2+^, Mg^2+^*_o_*—extracellular Mg^2+^, Kir—inward rectifier K^+^ channel, Ca_V_—voltage-gated Ca^2+^ channel, Na_V_—voltage-gated Na^+^ channel, BK—large conductance Ca^2+^-activated K^+^ channel, SK—small conductance Ca^2+^-activated K^+^ channel, P2XRs—purinergic P2X receptors, ATP—adenosine triphosphate, NMDAR—N-methyl-D-aspartate Receptor, Gly—glycine, Glu—glutamate, Asp—aspartate, GABA_A_R—type A Gamma-Amino Butyric Acid Receptor, [Mg^2+^]*_o_*—concentration of extracellular Mg^2+^, nAChR—nicotinic ACh receptor; red arrow—blocking action, green arrow—stimulating action, blue arrow—modulating action (surface charge screening), orange arrow—modulating action (phosphorylation), magenta arrow—modulating action (free ATP balance), gray arrow—unknown mode of action.

Figure 2 presents a visual summary of all major voltage-gated and ligand-gated ion channels in the nerve cell membrane, regulated by Mg^2+^.

Underlying mechanisms of Mg^2+^*_i_* and Mg^2+^*_o_* effects on neuronal ion channel function are concisely presented in Table 1.

### 2.3. Other Mechanisms Contributing to Electrophysiological Effects of Mg^2+^ in Nerve Cells

All the interactions described evidence the critical Mg^2+^-dependent modulation of neuronal voltage-gated and ligand-gated ion channels. Some other mechanisms additionally contribute to the manyfold neuroactive effects through which Mg^2+^ interferes with the regulation of intrinsic nerve cell membrane excitability, ionic mechanisms, and chemical and electrical synapses in the brain. For example, ion channels gated by other types of signals can also be Mg^2+^-sensitive, such as some mechanosensitive and temperature-gated channels (e.g., transient receptor potential melastatin type 7–TRPM7 channel) [176], gap junction channels (e.g., connexin type 36–Cx36 channel) [177], intracellular ion channels (e.g., RyR channel) [178], and finally, the activity of the Na^+^/K^+^ pump is also dynamically regulated by Mg^2+^ as an essential cofactor of the Na^+^/K^+^-ATPase [179]. Mg^2+^ is also involved in regulating certain subthreshold pacemaker ion currents in central neurons. Although Mg^2+^ is not the main regulator of Na_V_ channels, it can block the non-inactivating fraction of current through these channels–the persistent Na^+^ current (I_NaP_) active in many pacemaker neurons [80]. Aside from affecting I_NaP_, Mg^2+^ also helps regulate neuronal subthreshold pacemaker current through the hyperpolarization-activated cyclic nucleotide-gated (HCN) channels–the I_HCN_ current. While Mg^2+^ is not their primary regulator, it can indirectly influence HCN channel function by interacting with the intracellular environment (including cyclic nucleotides and other ions such as K^+^ and Na^+^) [180].

## 3. Discussion

Magnesium is the fourth most abundant cation in the body and participates in the regulation of important processes in excitable tissues, such as membrane electrolyte flux and antagonism with Ca^2+^. Mg^2+^ is responsible for pivotal regulatory effects on voltage-gated and ligand-gated ion channels, determining cellular excitability and signal transduction. Taking into account inevitable electrolyte imbalances developing during ischemic or traumatic insults, both in the heart and the nervous system, it is important to recognize pathways that involve Mg^2+^ and lead to the disruption of membrane electrical properties and cell function.

Mg^2+^*_i_* essentially carries the inward rectification of K^+^ currents through Kir channels during membrane depolarization, since it enters the cytoplasmatic and transmembrane regions of the channel pore, binds to negatively charged amino acid residues in the pore lining, thereby blocking outward current flow. Mg^2+^*_o_* can exert a similar blocking effect, which is contingent on channel inactivation and direct blocking action, also by targeting negatively charged sites in the extracellular channel domains. The effects that Mg^2+^*_o_* and Mg^2+^*_i_* elicit on Kir channels can modify the resting membrane potential and cellular excitability, causing changes in normal cardiac rhythm, neurological disorders such as epilepsy or ataxia, and glial disorders or sensorineural hearing loss.

Depletion of Mg^2+^ weakens the voltage-dependent Kir channel block, preventing excitable cells from restoring [K^+^]*_i_* and maintaining [K^+^]*_o_* in the physiological range after APs. In return, the cells experience excessive depolarization, hyperexcitability, and late repolarization, which can be seen in cardiomyocytes [181]. Kir2.1 channelopathy stemming from *KCNJ2* mutation manifests as Andersen–Tawill syndrome, where this channel is nonfunctional, and in addition to distinctive craniofacial features, patients suffer from periodic paralysis and long QT syndrome (LQT). Hyperexcitability in skeletal muscles leads to cramps and myotonia, episodically interrupted with muscle weakness, while the prolonged plateau phase of cardiac AP destabilizes membrane potential and can cause severe arrhythmias [181]. Since hypomagnesemia can exacerbate symptoms, administration of Mg^2+^ can be corrective for both the electrolyte imbalance and membrane stabilization. Severe Mg^2+^ deficit causes acquired LQT, which can be complicated with malignant arrhythmias, so urgent infusion of magnesium sulphate is indicated in these cases. Some Kir2.1 channel mutations are considered to be involved in retinopathy of prematurity, isolated arrhythmia with LQT phenotype (Thr 305 Ala substitution), or paroxysmal atrial fibrillation or ventricular tachycardia in patients with short QT syndrome (SQT3), where cardiac AP duration is shortened as well as the QT interval [14]. Mutations of *KCNJ10* encoding the Kir4.1 channel leads to epileptic seizures, ataxia, sensorineural hearing loss and renal tubulopathy (EAST syndrome) [14,181]. Functional Kir4.1 channels balance extracellular K^+^ after neuronal firing, maintain endolymph [K^+^] and mediate reabsorption and electrolyte balance in renal tubules, while mutant channels become unstable, lose their control of the current, leading to EAST syndrome.

Elevated physiological or supraphysiological [Mg^2+^]*_i_* directly inhibits permeation through Ca_V_ channels in a voltage-dependent manner. The reduction in current flow is modulated by Ca_V_ channel phosphorylation, considering an interplay of channel gating kinetics shift due to Mg^2+^-induced phosphorylation and phosphorylation-induced channel susceptibility to Mg^2+^*_i_* blocking action. Mg^2+^*_i_* competes with Ca^2+^–CaM for binding sites on Ca_V_ channel regulatory domains, affecting CDI and VDI of overstimulated cells. Mg^2+^*_o_* directly blocks Ca_V_ channels, an effect which is potentiated with membrane hyperpolarization, and modifies channel gating kinetics through surface charge screening. Ca_V_ channels play a crucial role in excitation–contraction coupling in every muscle type, drive synaptic neurotransmitter release, support gene transcription, and plasticity. Changes in [Mg^2+^] can subsequently modify the effectiveness of Ca^2+^-dependent processes, leading to life-threatening cardiovascular or neurologic conditions.

As Mg^2+^ can affect vascular smooth muscle tone and regulate peripheral vascular resistance, this indirectly modifies blood pressure (BP). Acting as a blocker of Ca_V_1 channels, Mg^2+^ reduces Ca^2+^ inward currents and myogenic tone, facilitating vasodilation. Depletion of Mg^2+^ severely impairs this effect, leading to vasoconstriction and hypertension [4]. On the other hand, diminished blocking of the Ca_V_1 channels in cardiomyocytes due to hypomagnesemia enhances Ca^2+^ influx and prolongs the AP plateau phase, delaying repolarization. Consequently, hypomagnesemia can trigger an acquired LQT [4]. Mutations of the Ca_V_1.1 or Na_V_1.4 channels are associated with hypokalemic periodic paralysis, a neurologic condition presenting with hypotonia and transient intervals of local or generalized paresis or paralysis [44] due to long-lasting membrane depolarization leading to Na_V_ channel inactivation and loss of muscle excitability. Ca_V_1.2 channels, highly expressed in hippocampal neurons, participate in long-term synaptic plasticity through the formation of spatial and fear memory and emotional behavior [41,44]. Dysregulation of these channels is related to forms of schizophrenia, bipolar affective disorder, and anxiety- and depression-like behaviors [41]. Mental disabilities, autism spectrum disorders, and severe cardiac arrhythmias are to some extent connected with Ca_V_1.2 channel mutations [41]. Gain-of-function mutation of Ca_V_1.2 in Timothy syndrome reduces the channel’s CDI and VDI, leading to excessive influx of Ca^2+^ and delayed cell repolarization, which destabilizes the cell membrane. LQT and neuronal hyperexcitability elicit severe arrhythmias and intellectual disability or autism in patients carrying this mutation [44]. Mutant or dysfunctional Ca_V_1.3 channels can lead to SAN dysfunction–bradycardia, arrhythmias, and sensorineural hearing loss due to insufficient Ca^2+^*_i_* handling. Interestingly, Ca_V_1.3 channels are an important gateway of [Ca^2+^]*_i_* increase in spontaneously active *substantia nigra* DA neurons, which are degenerated in Parkinson’s disease. DHP and related drugs–Ca_V_ channel blockers are commonly used to treat hypertension, but can be repurposed for the treatment of neuropsychiatric disorders, autism spectrum disorders, and neuroprotection in Parkinson’s disease [44]. Given its mechanism of action, it is conceivable that low [Mg^2+^]*_o_* can lead to increased Ca_V_ channel activity, leading to overexcitation of cells and manifesting similarly to disorders of different backgrounds with excessive Ca_V_ channel activation. Mg^2+^*_o_* blocks Ca_V_2 channels on presynaptic nerve endings, which modulates neurotransmitter release. It is plausible that low [Mg^2+^]*_o_* alleviates this blocking action and facilitates the release of neurotransmitters, which in turn overstimulates postsynaptic cell membranes. Gain-of-function Ca_V_2.1 channel mutation is linked to familial hemiplegic migraine, while loss-of-function mutation is connected with a form of episodic ataxia [42]. Although some Ca_V_2 channel mutations have subtle presentation, a gain-of-function mutation to Ca_V_2.2 channels can contribute to pain hypersensitivity or some forms of seizures [44]. In addition to its adjuvant analgesic effect mediated through NMDARs block, Mg^2+^*_o_* (magnesium sulphate) supports intraoperative neuromuscular blockade by reducing presynaptic Ca_V_2 channel activity, thus suppressing ACh release in the motor end plate [182]. Neurotic disorders are a group of functional psychiatric disorders encompassing diverse symptomatology such as hyperexcitability, anxiety, panic, and phobic reactions, attention deficits, and sleep disorders, among others. Hyperactivity of glutamatergic neurotransmission–increased synthesis and/or presynaptic Glu release is one of the mechanisms underlying these disorders. Mg^2+^*_o_* reduces glutamatergic neurotransmission by blocking Ca_V_2 channels on presynaptic neurons, alleviating anxiety, panic, and phobic reactions, and ameliorating sleeping deficits [183]. Mutations of the Ca_V_3.2 are linked to generalized absence epilepsies and idiopathic generalized epilepsies, as they increase neuronal firing by reducing the threshold for rebound excitation. Inhibition of Ca_V_3 channels protects from hyperexcitability disorders, such as absence epilepsy and certain forms of pain [42]. Additionally, mutations of all Ca_V_3 channels have been linked to autism spectrum disorder [42].

Mg^2+^*_i_* acts as a direct, rapid Na_V_ channel blocker, leading to flickering currents, but also modulates channel activity through channel phosphorylation. Elevated [Mg^2+^]*_o_* reduces Na_V_ channel conduction in both concentration-and voltage-dependent manner, alters channel voltage sensing with surface charge screening, and, interestingly, is proposed to have the ability to depolarize the cell by traversing the closed channel by quantum tunneling. It is evident that minute changes of [Mg^2+^]*_i_*, even within the physiological range, can be detrimental to the cell’s electrical activity. Na_V_ channels are essential in the initiation and propagation of APs in excitable cells. Perturbations in [Mg^2+^] can provoke severe, urgent disruptions of the fine-tuned cellular electrical activity, such as cardiac arrhythmias and bradycardias, muscle weakness, and epileptic seizures.

Mg^2+^*_o_* binds to the surface of skeletal muscle cell membrane, contributing to the electrical field. Na_V_ channels are sensing and affecting their activation. Mg^2+^ deficiency alters the voltage dependence of Na_V_ channel activation, allowing the channel to react to smaller membrane depolarization, leading to muscle spasms. Mutations of Na_V_1.1, Na_V_1.2, Na_V_1.3, or Na_V_1.6 are responsible for inherited epilepsy syndromes as inhibitory interneurons lose their tone, leading to hyperexcitability in the CNS [184]. *SCN4A* gain-of-function mutation encoding Na_V_1.4 channels can disable channel inactivation so that I_NaP_ in skeletal muscles elicits repetitive AP firing in some forms of myotonia or induces electrical silence in periodic paralysis [71,72]. Low [Mg^2+^]*_o_* may exacerbate myotonia in patients carrying *SCN4A* mutation, but supplementation with magnesium helps reduce weakness and myotonia [185]. Mg^2+^*_i_* can reduce Na_V_ channel conductance of central neurons in a voltage- and concentration-dependent manner [73], so conditions where [Mg^2+^]*_i_* is low increase endogenous neuronal excitability, leading to epileptiform activity. Furthermore, gain-of-function *SCN5A* mutation encoding Na_V_1.5 channel abrogates fast channel inactivation, impairs channel closure, and prolongs AP due to I_NaP_ [184], eliciting LQT3 syndrome [71,72], while loss-of-function mutation in Brugada syndrome desynchronizes conduction in the ventricles [72,184]. Mutations of *the SCN8A gene encoding the* Na_V_1.6 channel are associated with cognitive deficits and susceptibility to bipolar disorder [184]. While the Na_V_1.6 channel is one of the most abundant Na^+^ channels in the CNS and PNS, responsible for repetitive neuronal firing due to resurgent currents [186], current evidence does not indicate a direct Mg^2+^ modulatory effect on these channels in central neurons. Furthermore, it is plausible that disturbances in Mg^2+^ homeostasis might indirectly alter neuronal excitability by influencing channels’ firing patterns. Sensory neurons bearing Na_V_1.7 channel mutations become hyperexcitable, leading to erythromelalgia, a rare vascular neuropathic pain disorder characterized by erythema, local hyperthermia, and burning pain in extremities. Na_V_1.7, Na_V_1.8, and Na_V_1.9 channels are present in DRG neurons, and they are important for the transportation of pain signals [184]. Na_V_1.8 channels mediate most of the inward Na^+^ currents during APs, and their mutations lead to abnormal firing of DRG neurons [184].

BK channels are important for the negative feedback control of Ca^2+^ influx and excitability in the nervous and cardiovascular systems, as they colocalize with Ca_V_ channels to form nanodomains [48] and form BK-GluN1 complexes with NMDARs [187]. Activation of BK channels in response to increasing [Ca^2+^]*_i_* decreases AP duration, enhances hyperpolarization potentials, limits neurotransmitter release, and time for Ca^2+^ influx. The BK-GluN1 complex has been described in the hippocampus, cerebellum, cortex, thalamus, and striatum, and it is proposed that the BK channel α-subunit S0–S1 loop interacts with the C-terminal domain of NMDAR. Activation of NMDAR by glutamate elicits outward currents through BK channels in dentate gyrus granule cells [187] and pyramidal neurons in the neocortex [188].

Dysfunction of BK channels is implicated in epilepsy, fragile X syndrome, intellectual disability, autism, movement disorders, and chronic pain [189]. A myriad of loss-of-function and gain-of-function mutations of the *Slo1* (*KCNMA1*) gene generated in animal models help understand the role of BK channels in the etiopathogenesis of these disorders. The D434G mutant (Asp 434 Gly substitution) murine model shows generalized seizures and paroxysmal dyskinesia, most likely due to hyperexcitability of cortical and Purkinje neurons, as well as increased sensitivity of BK channels to Ca^2+^*_i_* [190]. A N995S mutation (Asn 995 Ser substitution) in the RCK2 domain that increases BK channel currents due to increased voltage sensitivity underlies paroxysmal non-kinesigenic dyskinesia [191]. Deletion of the *KCNMB4* gene coding the β4 subunit [192] increases firing in hippocampal dentate granule cells, leading to temporal lobe epilepsy. Alteration in the regulatory β3-subunit as a result of a single base pair deletion in the *KCNMB3* gene is associated with generalized epilepsy [193]. The BK channel G354S mutation (Gly 354 Ser substitution), which reduces channel conductance and ion selectivity, has been linked to congenital progressive cerebellar ataxia with cognitive impairment [194]. Single-nucleotide polymorphism in genes encoding α-subunit and β2-subunit (rs16934131 and rs637454, respectively), is associated with the risk of Alzheimer’s disease [195,196]. Release of excitatory neurotransmitters and calcitonin gene-related peptides from the trigeminal caudate nucleus is inhibited by BK channels, so they can be a potential target for the therapy of migraines [197]. The BK channels’ role in regulating neuronal firing and cell excitation is dependent on Ca^2+^*_i_* and membrane depolarization. Depletion of Mg^2+^*_i_* dampens BK channel activation, leaving neurons unprotected from overexcitation by increasing [Ca^2+^]*_i_*. BK channels are also an important regulator of smooth muscle cell excitability and vascular muscle tone since they mediate outward K^+^ currents. Mg^2+^*_i_*, in cooperation with Ca^2+^*_i_*, facilitates BK channel activation and reduces muscle cell excitation, leading to its relaxation and vasodilation. Vascular smooth muscle cells can have membrane microdomains where BK channels closely cooperate with Ca_V_ channels to regulate Ca^2+^*_i_* excitatory effect. Elevated [Mg^2+^]*_o_* is rare, but it can mediate a complex interplay between Mg^2+^*_o_*-mediated suppression of Ca_V_ channel inward Ca^2+^ current and Mg^2+^*_i_*-mediated activation of BK channel with large outward K^+^ current, leading to a decrease in smooth muscle membrane excitability and vasodilation [4]. It is then understandable why hypomagnesemia with both [Mg^2+^]*_o_* and [Mg^2+^]*_i_* reduction contributes to vasoconstriction and development of hypertension.

SK channels regulate membrane AHP, which is important for the regulation of intrinsic neuronal excitability and AP firing rate. Correspondingly to BK channels, they are located in the vicinity of Ca_V_ channels, NMDARs, or nAChRs and modify cell excitability upon activation by one of these ports of Ca^2+^ entry. In this manner, they affect synaptic plasticity in neurons and lead to AP completion in cardiomyocytes.

As SK channels are present on DA neurons in the midbrain, they control their firing patterns by counteracting the excitatory Ca^2+^ influx. Pathogenesis of Parkinson’s disease is related to these channels, which coordinate their activity with Ca_V_ channels to form patterns of DA release [198,199]. As the burst activity of these neurons releases DA [200], block of SK channels by Mg^2+^*_i_* or channel blockers (apamin) may alleviate symptoms of Parkinson’s disease [201,202]. This might be controversial since unregulated Ca^2+^ influx through activated NMDARs and Ca_V_ channels in DA neurons leads to their excitotoxic death and overall loss in the *substantia nigra*. Conversely, some studies show that SK channel activation results in afterhyperpolarization [203] in DA neurons, it reduces excitotoxicity [204] and enhances or preserves DA synthesis to mitigate long-term motor disorders in Parkinson’s disease. These contradictory findings are best explained by the effect of SK channel modulation in line with the stage of Parkinson’s disease. The gene *KCNN3* encoding SK3 channels contains a sequence of trinucleotide CAG repeats, which is associated with schizophrenia and bipolar disorders [205,206]. Animal models with *KCNN3* gene mutation present with suppressed SK3 channel activity in DA neurons, diminished counterbalance to NMDARs activation, which increases burst firing and release of DA, leading to attention deficits and sensory-motor alterations in behavior [207]. Recordings from Jurkat T cells expressing human mutant SK3 channel confirm the reduced channel activity previously described in animal models [208]. Moreover, a mutation of the CK3 channel resulting in the deletion of its N-terminal region was identified in patients with schizophrenia [209]. Mutations of SK channels have also been related to epilepsy. Expression of these channels in an animal model of epilepsy is shown to be significantly reduced [210]. Furthermore, *in vitro* [211], and *in vivo* [212,213] studies demonstrate that the use of SK channel activators reduces epileptiform activity. Activation of SK channels in atrial cardiomyocytes repolarizes them and reduces AP duration, acting as a negative feedback mechanism to Ca^2+^ influx during APs. Patients suffering from chronic atrial fibrillation exhibit a prominent shortening of AP duration, and it is known that AP shortening and prolongation may facilitate atrial arrhythmias in a similar fashion [118]. Expression of SK2 and SK3 channels in patients with chronic atrial fibrillation is reduced compared to healthy individuals [214].

NMDA receptors are inhibited by Mg^2+^*_i_* binding to GluN1 subunit amino acid residues deep within the channel pore, where Mg^2+^ can be “locked-in” by other cations binding distally in the channel. Mg^2+^*_o_* plays a crucial role in stabilizing cell excitability through voltage-dependent block of the NMDAR channels, binding to Asn residues (N-site) in adjacent GluN2 subunits and/or to TM1 and TM4 segments or TM2/TM3 linker in GluN2B subunits. NMDARs are crucial for synaptic plasticity, learning, and memory, but also orchestrate excitotoxicity in neurons in hypomagnesemia, leading to neurodegeneration in chronic settings or seizure activity in more acute cases. Hypermagnesemia impedes LTP as NMDARs are more resilient to depolarization, leading to learning deficits.

Mg^2+^*_o_* affects central excitatory glutamatergic synapses by modulating the activity of NMDARs, which at resting membrane potentials are blocked by these ions. A complex cascade of Glu-induced AMPAR activation and membrane depolarization removes the voltage-dependent Mg^2+^*_o_* block of NMDAR concomitantly stimulated by Glu and Gly and allows mostly an inward current flux through the NMDAR channel to elicit activation of neurons. The complex interplay of mechanisms occurring at low [Mg^2+^]*_o,_* which results in neuron overstimulation, is led by heightened NMDAR activation and excitotoxicity, where accumulation of Ca^2+^*_i_* expedites neuronal death. It is demonstrated that magnesium sulphate enters the cerebrospinal fluid and brain after systemic administration in an animal model. Consequent [Mg^2+^]*_o_* increase in the CNS disturbs binding of agonists (glutamate and glycine) to NMDARs in the hippocampus and cerebral cortex, providing insight into Mg^2+^ central anticonvulsant effect [215,216]. Mg^2+^*_o_* can disrupt the pathophysiological mechanism of NMDARs hyperactivity occurring in ischemic or traumatic brain injury that causes excitotoxic neuronal death [138,145], and it has been proven that magnesium therapy helps cognitive performance after brain injury. NMDAR activation can trigger cortical spreading depression (CSD), an electrical disturbance associated with seizures, migraine aura, and traumatic and ischemic brain injury, where localized intense depolarization of neurons and glial cells is followed by membrane ion flux change leading to an increase of [Ca^2+^]*_i_* while [Ca^2+^]*_o_* decreases [217]. CSD mechanism releases large amounts of Glu, causing a strong wave of depolarization spreading through the neighboring areas of the CNS. Hypomagnesemia can facilitate NMDAR activation and potentiate Glu effects, thus taking part in CSD initiation and spreading. In addition, physiological [Mg^2+^]*_o_* maintains Ca^2+^*_i_* balance by stabilizing NMDARs and blocks excessive Ca^2+^ influx and excitotoxicity, thus suppressing the production of substance P, one of the culprits in migraine pathogenesis [218]. Sensorineural hearing loss due to noise trauma is a result of NMDARs overstimulation in auditory neurons by large amounts of Glu released in ribbon synapses from cochlear inner hair cells reacting to higher amplitude of basilar membrane oscillation [219]. Several animal studies [220,221] demonstrate low [Mg^2+^] in the inner ear perilymph upon noise exposure and noise-induced hearing loss (NIHL), confirming the role of NMDAR involvement. In addition, studies on humans show that decreased serum [Mg^2+^] increases susceptibility to NIHL [222] and that magnesium supplementation can have therapeutic and prophylactic effects [223]. Another important role of Mg^2+^ is the prevention of NMDAR-mediated central sensitization following repetitive nociceptive inputs and the attenuation of pain hypersensitivity. Increase in neuronal [Ca^2+^]*_i_* upon repetitive stimulation is pivotal for central sensitization, but Mg^2+^*_o_* can help regulate this Ca^2+^ imbalance by antagonizing NMDARs, which reduces Ca^2+^ influx into neurons [224,225]. Considering its impact, Mg^2+^ (magnesium sulphate) is used as an adjuvant therapy for intra- and post-operative pain management and reduces analgesic requirements [226,227]. Interestingly, animal models exposed to dietary reduction in Mg^2+^ intake spanning a year present with atrophy and loss of *pars compacta substantia nigra* DA neurons [228,229,230]. Consistent with animal studies, a significant reduction of [Mg^2+^]*_i_* has been observed in patients with Parkinson’s disease [231]. Similarly, [Mg^2+^]*_o_* is reduced in the serum [232] and brain tissue (entorhinal cortex, hippocampal CA region, and *globus pallidus*) [233] of patients with Alzheimer’s disease. It is conceivable that Mg^2+^*_o_* might play a neuroprotective role in Parkinson’s disease and Alzheimer’s disease by modulating NMDAR activity [8], but these effects need to be studied further. Elevated activity of Glu stimulation i.e., Glu overstimulation of postsynaptic NMDARs, is one of the central mechanisms behind neurotic disorders. Most patients suffering from these disorders present with reduced intraerythocyte [Mg^2+^]*_i_* and [Mg^2+^]*_o_*. Mg^2+^*_o_* effects on Glu transmission are dual–suppression of presynaptic Glu release due to Ca_V_ blocking and direct competition with Ca^2+^ for NMDARs [183]. In light of these mechanisms, it is understandable that hypomagnesemia can increase neuronal vulnerability to various types of stressors in the development of neurotic disorders. More severe psychoses–acute schizophrenic episodes and bipolar disorder are accompanied by [Mg^2+^] decrease in the patient’s cerebrospinal fluid [234] or red blood cells [235], which steadily increases upon implementing antipsychotic therapy. Additionally, low plasma [Mg^2+^] is also present in patients with chronic schizophrenia [236]. It is proposed that Mg^2+^ reduces Glu release and Glu effects on NMDARs, as well as augments the activity of the GABAergic system.

Purinergic milieu *in vivo* varies across tissues in physiological states (homeostasis) and under pathophysiological conditions (inflammatory reaction, ischemia, etc.), depending on the concentrations of extracellular ATP and Mg^2+^*_o_*. Mg^2+^-dependent P2XR channel modulation contributes to the spatiotemporal tuning of P2XR signaling in the CNS, cardiovascular, and immune system, emphasizing Mg^2+^ status as a meaningful axis of variability in P2XR function in health and disease. Reviews synthesize P2XR ion channel dysfunctions contributing to nerve signaling, pain, inflammation, and different disease phenotypes. Several pathogenic variants of P2XRs and their disease associations have been documented, with the clearest human mutation–phenotype link in P2X2-related hearing loss. Broader receptor–disease connections are established by expression and functional studies across systems. Mutations of the human P2X2 receptor have been implicated in hereditary hearing loss, as these mutant variants alter P2X2 channel signaling in cochlear pathways, aligning with their role in auditory processing. Recent reports detail hearing loss-associated mutations affecting P2X2 channel function. Other than P2X2R association with auditory function, receptor–disease associations have been identified for other specific P2XR subtypes, P2X1 with platelet aggregation, P2X3 with asthma and sensory signaling, and P2X7 with vascular inflammation and immune responses. P2XR channelopathies are also being investigated in neurodegenerative conditions [237]. A study on murine *substantia nigra* DA neuron culture shows that P2X7Rs are directly involved in extracellular ATP-induced cell swelling and death [238]. Irregular activation of P2XRs mostly reported in microglial cells, can lead to a spectrum of mechanisms underlying Parkinson’s disease, Alzheimer’s disease, amyotrophic lateral sclerosis, and multiple sclerosis [239], yet diverse evidence of neuronal P2XR channelopathies in the etiopathogenesis of these diseases is lacking.

Concerning the effects of Mg^2+^ disorders, evidence directly connecting alterations of Mg^2+^ status to human P2X channelopathies is limited. In a context where a mutant receptor relies more on free ATP for efficient gating (P2X2, such as behavior), higher Mg^2+^*_o_* (favoring Mg-ATP^2−^) could reduce activation efficacy, potentially worsening functional deficits, and conversely, lower Mg^2+^*_o_* could increase free ATP availability and enhance receptor activation. More data are available on dysfunctions of standard, naturally occurring P2XR channel variants that arise from Mg^2+^ imbalances, which can perturb purinergic signaling in several ways, by shifting ATP speciation and allosteric modulation, producing subtype-dependent changes in P2XR activation, binding–gating coupling, and downstream cation flux. High Mg^2+^*_o_* elevates Mg-ATP^2−^ levels and reduces free ATP levels, generally diminishing activation efficacy in subtypes like P2X2, while leaving binding relatively preserved; P2X3 subtype may show decreased activation with paradoxically increased binding, while heteromeric P2X2/3 displays mixed effects. Functional consequences include attenuated cation influx and altered desensitization dynamics, contingent on subtype composition [160,161].

Inhibitory effect of Mg^2+^*_o_* on GABA_A_R channels is concentration-dependent, as physiological Mg^2+^*_o_* levels allow maximal inward current flow through the channel, while higher concentrations reduce current amplitude and increase GABA_A_ receptor susceptibility to Cl^−^ channel blockers. GABA_A_Rs mediate inhibitory neurotransmission in the CNS and balance the excitatory signaling mediated by AMPARs and NMDARs. Mg^2+^ and GABA together achieve a neuroprotective synergistic effect through GABA_A_Rs activation. Mg^2+^ depletion can reduce GABA_A_R efficacy, leading to hyperexcitability, anxiety, insomnia, and seizure activity, whereas hypermagnesemia can amplify the intricate GABAergic inhibition, leading to cognitive impairment, sedation, and muscle weakness.

Acting as a GABAAR agonist, physiological [Mg^2+^]*_o_* causes neuron hyperpolarization, mediating Cl^−^ influx and protecting it from excessive synaptic excitation from other sources. Mg^2+^*_o_* leads to concentration-dependent reversible suppression of epileptiform activity in hippocampal neurons induced by a GABA_A_R antagonist, bicuculine, through a direct block of Ca_V_ channel-mediated currents in postsynaptic neurites, as well as in presynaptic membranes to modulate synaptic transmission. Additionally, Mg^2+^*_o_* alters Na_V_ channel activation while stimulating GABA_A_Rs [240]. Neurotic disorders can stem from a decrease in GABAergic activity, and Mg^2+^ helps reduce some of the symptomatology by enhancing the function of GABA-mediated neurotransmission and increasing GABA release [183]. The GABAergic system, which is suppressed in patients with schizophrenia, can be reactivated by increasing neuronal Mg^2+^ following antipsychotic drug therapy [164,241].

Mg^2+^*_o_* inhibits the activity of nACh receptors in a two-fold action, both being voltage-dependent. Interaction between Mg^2+^*_o_* and the binding sites in the channel pore lining impedes current flow, while interaction with the outer membrane surface through surface charge screening alters membrane voltage and channel gating properties. Nicotinic ACh receptors play a crucial role in neuromuscular signaling and motor control, autonomic nervous system output, and cognitive processing. Hypomagnesemia alleviates nAChRs from its blocking action, leading to muscle spasm, neuronal hyperexcitability, and autonomic nervous system hyperactivity. Conversely, high Mg^2+^ levels promote the attenuation of nAChR activity, leading to a suppression of electrical activity in most of the excitable tissues expressing these receptors.

By interacting with nAChRs, Mg^2+^ acts as a modulator of ion fluxes–centrally it reduces nAChR conductance, and peripherally it blocks the neuromuscular junction. Mg^2+^ potentiates intraoperative neuromuscular block elicited by known non-depolarizing nACh blockers (d-tubocurarine or vecuronium), possibly through bimodal action in the motor endplate–diminution of ACh-induced depolarizing action caused by nAChR block and suppression of ACh release from axon terminals due to Ca_V_ channel block [242,243].

## 4. Conclusions

Mg^2+^ ion is a homeostatic agent and one of the simple biological regulators of cell excitability. Both Mg^2+^*_i_* and Mg^2+^*_o_* exert pleotropic impacts on elements of synaptic and non-synaptic cell excitability. Their effects are most pronounced in central neurons, where a multitude of mechanisms contribute to the complex regulation of ion channel function, allowing Mg^2+^ to fine–tune cell membrane ion transport. Appreciating these cellular and tissue processes in the brain at which Mg^2+^ is interposed to maintain normal excitability as a vital body function can help us better understand and treat clinical conditions with neurological dysfunctions due to hyper-or hypoexcitability caused by disorders of Mg^2+^ homeostasis.

Magnesium ions are an integral part of the intricate mechanisms of membrane excitability control. Studying their impact on the function of voltage-gated and ligand-gated ion channels shows how delicate changes in the internal balance of Mg^2+^ provoke severe disruptions in cell excitability, function, and morphology. Despite extensive research already undertaken, some of the mechanisms of Mg^2+^*_i_* and Mg^2+^*_o_* effects on ion channels’ functions are still elusive and unknown.

## 5. Future Directions

Development of more precise imaging techniques, in silico molecular modeling, optogenetics, and ultra-high resolution electrophysiological recording combined with channel mutagenesis, warrants further discoveries concerning Mg^2+^-regulated ion channels and, more importantly, new targets for magnesium-based therapy for some inherited and acquired ion channel disturbances and subsequent clinical conditions.

## Figures and Tables

**Table 1:** Underlying mechanisms of Mg^2+^*_i_* and Mg^2+^*_o_* effects on ion channels in central neurons.

Channel Type	Mg^2+^*_i_* Effects	Mg^2+^*_o_* Effects	Functional Consequences	References
Kir	Inward rectification via blocking outward K^+^ current; binding to negatively charged residues in the CTD and TM2 domain	Outward K^+^ current block by binding to the extracellular channel domain; depends on channel inactivation	Alters resting membrane potential and excitability; linked to arrhythmia, epilepsy, hearing loss, and hypertension	Mg^2+^*_i_* effects [11,12,13,14,15,16,17,18,19,20,21,22,23,24,25,26,27,28,29,30]; Mg^2+^*_o_* effects [11,16,19,20,24,31,32]
Ca_V_	Voltage-dependent channel pore block; channel gating modulation via phosphorylation and inactivation of phosphorylated channel; competition with Ca^2+^-CaM binding to EF-hand motif	Direct voltage- and concentration-dependent blocking action, enhanced by membrane hyperpolarization; surface charge screening alters channel gating kinetics	Disrupts excitation-contraction coupling, neurotransmission, and gene expression; may cause cardiovascular or neurological disorders	Mg^2+^*_i_* effects [54,55,56,57,58,59,60,61,62,63,64,65]; Mg^2+^*_o_* effects [44,57,61,66,67,68,69,70]
Na_V_	Rapid direct channel block; competitive nature of the blocking action; modulation of channel gating via phosphorylation	Voltage-and concentration-dependent block; surface charge screening alters channel voltage sensing; may cause depolarization via quantum tunneling	Bradycardia, arrhythmias, muscle weakness, excitability disruption, and seizures	Mg^2+^*_i_* effects [72,73,74,75,76,77]; Mg^2+^*_o_* effects [78,79,80,81,82,83]
BK	Voltage- and concentration-dependent block; negative charge screening; channel activation	Concentration-dependent decrease in outward current; negative charge screening	Generalized seizures; temporal lobe epilepsy; risk of Alzheimer’s disease; paroxysmal dyskinesia; hypertension	Mg^2+^*_i_* effects [90,91,92,93,94,95,96,97,98,99,100,101,102,103,104,105,106,107,108,109,110,175] Mg^2+^*_o_* effects [91,92,99,111]
SK	Voltage- and concentration-dependent block; inward rectification of channel I–V relation	not discussed directly	Schizophrenia; bipolar disorder; susceptibility to Parkinson’s disease; atrial fibrillation	Mg^2+^*_i_* effects [119,120,121]
NMDAR	Voltage-dependent block; binding to GluN1 deep in the channel pore; entrapment by cations bound distally	Receptor stabilization via voltage-dependent block; GluN2 subunit binding (residues in TM segments); increase in receptor affinity to Gly	Alterations in excitation, excitotoxicity, seizures, neurodegeneration (low [Mg^2+^]*_o_*); alterations in plasticity, memory, LTP impairment (high [Mg^2+^]*_o_*)	Mg^2+^*_i_* effects [125,126,127,128,129,130,131,132,133,134,135,136,137]; Mg^2+^*_o_* effects [125,126,135,137,138,139,140,141,142,143,144,145,146,147,148,149,150,151,152,154,155,156,157]
P2XRs	no direct effect	Subtype-specific modulation of P2XR channel activity	Hereditary hearing loss; pain pathogenesis	Mg^2+^*_o_* effects [158,159,160,161,162]
GABA_A_R	not discussed directly	Concentration-dependent inhibition; high [Mg^2+^]*_o_* reduces Cl^−^ current and increases susceptibility to channel blockers	Complementary action with GABA in neuroprotection; hyperexcitability (low [Mg^2+^]*_o_*); sedation, cognitive impairment (high [Mg^2+^]*_o_*)	Mg^2+^*_o_* effects [163,164]
nAChR	Voltage-dependent channel block; alteration of channel gating; reduction in outward channel conductance	Direct voltage-dependent channel pore block; local surface membrane potential change alters channel gating	Hyperexcitability, muscle spasms (low [Mg^2+^]*_o_*); neuromuscular and autonomic activity suppression (high [Mg^2+^]*_o_*)	Mg^2+^*_i_* effects [169,171,172,174]; Mg^2+^*_o_* effects [167,168,169,170,171,172]

Mg^2+^*_i_*—intracellular Mg^2+^, Mg^2+^*_o_*—extracellular Mg^2+^, Kir—inward rectifier K^+^ channel, CTD—cytoplasmic domain, TM—transmembrane, Ca_V_—voltage-gated Ca^2+^ channel, CaM—calmodulin, Na_V_—voltage-gated Na^+^ channel, BK—large conductance Ca^2+^-activated K^+^ channel, SK—small conductance Ca^2+^-activated K^+^ channel, NMDAR—N-methyl-D-aspartate Receptor, P2XRs—purinergic P2X receptors, Gly—glycine, I–V—current-voltage, LTP—long-term potentiation, GABA_A_R—type A Gamma-Amino Butyric Acid Receptor, [Mg^2+^]*_o_*—concentration of extracellular Mg^2+^, nAChR—nicotinic ACh receptor.

## Data Availability

No new data were created or analyzed in this study. Data sharing is not applicable to this article.

## Abbreviations

The following abbreviations are used in this manuscript:

ER	endoplasmic reticulum
Mg^2+^	magnesium ions
Na^+^	sodium ions
K^+^	potassium ions
Ca^2+^	calcium ions
Cl^−^	chloride ions
Ca^2+^*_i_*	intracellular Ca^2+^
iMg^2+^	ionized Mg^2+^ fraction
Mg^2+^*_i_*	intracellular (inner) Mg^2+^
Mg^2+^*_o_*	extracellular (outer) Mg^2+^
[Mg^2+^]	concentration of Mg^2+^
[Mg^2+^]*_i_*	concentration of intracellular Mg^2+^
[Mg^2+^]*_o_*	concentration of extracellular Mg^2+^
ATP	adenosine triphosphate
ADP	adenosine diphosphate
VGC	voltage-gated channel
VSD	voltage-sensing domain
Kir	inward rectifier potassium channel
TM	transmembrane
CTD	cytoplasmic domain
MEL	murine erythroleukaemia
Asp	Aspartate (D)
Asn	Asparagine (N)
Glu	Glutamate (E)
Gln	Glutamine (Q)
Gly	Glycine (G)
Ser	Serine (S)
[K^+^]*_i_*	concentration of intracellular K^+^
[K^+^]*_o_*	concentration of extracellular K^+^
His	Histidine (H)
K_V_	voltage-gated potassium
KCNQ	potassium voltage-gated channel subfamily Q
PIP2	phosphatidylinositol 4,5-bisphosphate
EAG	ether-à-go-go
hERG	human ether-à-go-go related gene
Ca_V_	voltage-gated calcium
PD	pore domain
DHP	dihydropyridine
SAN	sinoatrial node
DHPR	dihydropyridine receptor
AP	action potential
HVA	high-voltage activated
LVA	low-voltage activated
[Ca^2+^]*_i_*	concentration of intracellular Ca^2+^
[Ca^2+^]*_o_*	concentration of extracellular Ca^2+^
VDI	voltage-dependent inactivation
CDI	Ca^2+^-dependent inactivation
IQ	isoleucine-glutamine
CaM	calmodulin
AID	α1-interacting domain
HEK	human embryonic kidney
Ala	Alanine (A)
Lys	Lysine (K)
DCT	distal C-terminal domain
PCT	proximal C-terminal domain
cAMP	cyclic adenosine monophosphate
PKA	protein kinase A
PP2A	phosphatase 2A
DRG	dorsal root ganglion
PC12	rat pheochromocytoma cells
Na_V_	voltage-gated sodium channel
CNS	central nervous system
PNS	peripheral nervous system
[Na^+^]*_i_*	concentration of intracellular Na^+^
[Na^+^]*_o_*	concentration of extracellular Na^+^
TTX	tetrodotoxin
IC_50_	half maximal inhibitory concentration
BK	large conductance Ca^2+^-activated potassium (channel)
NMDAR	N-methyl-D-aspartate Receptor
RyR	ryanodine receptor
PGD	pore-gate domain
RCK	regulator of K^+^ conductance
mSlo1	mouse Slo1
Cys	Cysteine (C)
G–V	Conductance–Voltage relationship
Arg	Arginine (R)
Trp	Tryptophan (W)
*I_g_*	transient gating current
Leu	Leucine (L)
SK	small conductance Ca^2+^-activated potassium (channel)
AHP	afterhyperpolarization
CaMBD	CaM binding domain
DA	dopamine, dopaminergic
LTP	long-term potentiation
I–V	Current–Voltage relationship
rSK2	rat SK2 (channel)
NMDA	N-methyl-D-aspartate
P2XR	purinergic P2X receptors
GABA	Gamma-Amino Butyric Acid
nAChR	nicotinic ACh receptor
iGluR	ionotropic glutamate receptor
ATD	amino-terminal domain
LBD	ligand-binding domain
AMPA	α-amino3-hydroxy-5-mathyl-4-isoxazole propionic acid
EPSC	excitatory postsynaptic current
AMPAR	α-amino3-hydroxy-5-mathyl-4-isoxazole propionic acid receptor
GABA_A_R	type A Gamma-Amino Butyric Acid Receptor
ECD	extracellular domain
ACh^+^	acetylcholine oxyanion
ACh	acetylcholine
TRPM7	transient receptor potential melastatine type 7 channel
Cx36	connexin type 36
I_NaP_	persistent Na^+^ current
HCN	hyperpolarization-activated cyclic nucleotide-gated channels
I_HCN_	ion current through HCN channels
LQT	long QT
SQT	short QT
EAST	epileptic seizures, ataxia, sensorineural hearing loss, renal tubulopathy
BP	blood pressure
CSD	cortical spreading depression
NIHL	noise-induced hearing loss
